# Current and Emerging Endovascular and Neurocritical Care Management Strategies in Large-Core Ischemic Stroke

**DOI:** 10.3390/jcm12206641

**Published:** 2023-10-20

**Authors:** Ibrahim Migdady, Phoebe H. Johnson-Black, Thabele Leslie-Mazwi, Rishi Malhotra

**Affiliations:** 1 Division of Critical Care Medicine, Albert Einstein College of Medicine, Montefiore Medical Center, Bronx, NY 10467, USA; 2 Department of Neurology, Albert Einstein College of Medicine, Montefiore Medical Center, Bronx, NY 10467, USA; 3 Department of Neurological Surgery, Albert Einstein College of Medicine, Montefiore Medical Center, Bronx, NY 10467, USA; 4 Department of Health Policy and Management, Mailman School of Public Health, Columbia University, New York, NY 10032, USA; 5 Department of Neurosurgery, Division of Neurocritical Care, UCLA David Geffen School of Medicine, Ronald Reagan Medical Center, Los Angeles, CA 90095, USA; phoebejohnson@mednet.ucla.edu; 6 Department of Neurology, University of Washington, Seattle, WA 98195, USA; tml01@uw.edu

**Keywords:** large-core infarcts, malignant edema, large-vessel occlusion, endovascular therapy

## Abstract

The volume of infarcted tissue in patients with ischemic stroke is consistently associated with increased morbidity and mortality. Initial studies of endovascular thrombectomy for large-vessel occlusion excluded patients with established large-core infarcts, even when large volumes of salvageable brain tissue were present, due to the high risk of hemorrhagic transformation and reperfusion injury. However, recent retrospective and prospective studies have shown improved outcomes with endovascular thrombectomy, and several clinical trials were recently published to evaluate the efficacy of endovascular management of patients presenting with large-core infarcts. With or without thrombectomy, patients with large-core infarcts remain at high risk of in-hospital complications such as hemorrhagic transformation, malignant cerebral edema, seizures, and others. Expert neurocritical care management is necessary to optimize blood pressure control, mitigate secondary brain injury, manage cerebral edema and elevated intracranial pressure, and implement various neuroprotective measures. Herein, we present an overview of the current and emerging evidence pertaining to endovascular treatment for large-core infarcts, recent advances in neurocritical care strategies, and their impact on optimizing patient outcomes.

## 1. Introduction

In the past two decades, the treatment of acute ischemic stroke has witnessed significant progress, mainly driven by the widespread use of recombinant intravenous tissue-plasminogen activator (tPA), the adoption of endovascular therapy (EVT), and advancements in the subacute-to-chronic phases of care. These developments have had a profound impact on patient outcomes, improving survival rates and reducing long-term morbidity. Consequently, there is a growing need for personalized approaches to acute and post-acute treatment in the hospital setting.

It is estimated that one-fourth of patients with large-vessel occlusion (LVO) strokes present with established large-core infarcts (LCI) [1]. Traditionally, patients with LCI were excluded from tPA and endovascular therapy trials due to concerns regarding the risks of hemorrhagic conversion, reperfusion injury, and the perceived limited benefits when a substantial portion of the brain had already experienced infarction. As a result, the primary focus of treatment for this patient subgroup has been on mitigating secondary injuries arising from cerebral edema, increased intracranial pressure, hemorrhagic conversion, and other in-hospital complications. Specialized neurocritical care has played a pivotal role in managing these patients, particularly in cases of complete middle-cerebral artery (MCA) infarcts, where early decompressive hemicraniectomy (DHC) demonstrates improved survival rates and functional outcomes [2]. Other commonly used management strategies for patients with LCI encompass meticulous blood pressure control, implementation of neuroprotective measures, identification and treatment of seizures, optimization of cerebral perfusion, support for collateral circulation of tissue-at-risk, and prevention of stroke recurrence.

Recent studies have revealed promising findings regarding the expanded use of EVT in specific subsets of patients with LCI and substantial volumes of salvageable tissue. However, it is important to acknowledge that EVT cannot reverse brain tissue death resulting from infarction but rather aims to reduce final infarct volume, which is a more relevant predictor of outcome than recanalization [3]. Therefore, patients with LCI undergoing thrombectomy remain at risk of secondary brain injury, necessitating attentive patient care focused on improving their outcomes.

In this article, we comprehensively review the latest evidence and practices pertaining to advances in endovascular and neurocritical care management of LCI, shedding light on the current scientific evidence and providing valuable insights for clinical practice.

## 2. Definition of Large-Core Infarct Ischemic Stroke

The volume of infarcted tissue can be measured on computed tomography (CT), CT-perfusion (CTP), magnetic resonance diffusion weighted imaging (MRI-DWI) or apparent-diffusion coefficient sequences (ADC) by applying the ABC/2 method [4], or through automated, commercially available CT and MRI analysis software [5]. The Alberta Stroke Program Early CT Score (ASPECTS), a score from 0 to 10 inversely correlating with the severity of MCA strokes, is also widely utilized, with ASPECTS < 6 regarded as an LCI. However, due to low inter-rater agreement, ASPECTS is increasingly being replaced by quantitative measurement of infarcted brain tissue using CT or MRI.

There is no agreed-upon definition of what constitutes an LCI; however, a strong relationship between initial infarct volumes and outcome has been well established. The cut-offs for the minimal infarct volume that constitutes a large infarct are derived from studies evaluating the impact of initial or final infarct volumes on clinical outcomes; the most common cut-off used in clinical trials ranges from 50 to 70 mL of infarcted brain tissue [6,7]. For example, in the pivotal DEFUSE-3 trial of thrombectomy for stroke within 6–16 h, patients with core infarct volume (measured on MRI-DWI) > 70 mL were excluded because prior studies had shown that initial MRI-DWI volume of infarcted tissue ≥ 70 mL results in poor clinical outcomes regardless of recanalization status [8]. In a post-hoc analysis of the Echoplanar Imaging Thrombolytic Evaluation Trial (EPITHET) of acute ischemic stroke patients imaged with serial MRI and randomized to tPA or placebo at 3 to 6 h after symptom onset, a benefit of tPA was seen with DWI lesions up to 25 mL, but not for DWI lesions > 25 mL, and patients with baseline DWI lesions > 65 mL had a very high rate of poor outcome, regardless of treatment group [9]. In one retrospective study of 37 patients with acute MCA infarction and proximal vessel occlusion (carotid-T segment or MCA mainstem) who underwent MRI-DWI and ADC imaging within 6 h of onset (before the era of EVT), an infarct volume > 82 mL was associated with a sensitivity of 87% and specificity of 91% for predicting progression to malignant cerebral edema [10]. In a meta-analysis of 38 studies (3278 patients), a parenchymal hypoattenuation >50% of the MCA territory on initial CT was associated with malignant cerebral edema (*n* = 420 patients; odds ratio [OR], 5.33 [95% CI, 2.93–9.68]) [11]. It is important to note that CTP tends to overestimate infarct volume compared to other modalities [12].

For the purpose of this review, discussions regarding LCI pertain to those defined by studies of EVT or DHC for malignant cerebral edema as >50–70 mL of infarcted tissue.

## 3. Thrombolysis for Large-Core Ischemic Stroke

Patients with LCI have been historically excluded from the most pivotal trials of intravenous tPA for acute stroke treatment within 4.5 h of symptom onset. In the first trial to establish efficacy of tPA in acute ischemic stroke (NINDS trial 1995), initial stroke volumes were not assessed or used in the inclusion criteria [13]. The first European Cooperative Acute Stroke Study (ECASS 1) trial was the first to establish the improved safety and efficacy of tPA when given to patients without extended infarcts on initial CT [14]. In the subsequent ECASS trials, patients were excluded from enrollment if they presented with severe stroke, defined as a National Institutes of Health Stroke Scale (NIHSS) score >25 or a stroke involving >1/3 of the MCA territory on initial CT due to a concern for increased risk of symptomatic intracerebral hemorrhage (sICH) [15]. As a result, LCI are currently part of the exclusion criteria for tPA in routine clinical practice and randomized clinical trials.

Because patients with LCI are systematically excluded from receiving tPA, substantial limitations in the literature pertaining to the safety and efficacy of IV tPA in LCI exist, and the exact infarct volume that increases risk of hemorrhagic conversion after IV tPA has not been thoroughly studied in randomized clinical trials and has only been evaluated in underpowered, retrospective studies. Recent studies have evaluated the safety of tPA in the era of EVT. In a retrospective analysis of 398 patients enrolled in the Stroke Thrombectomy Aneurysm Registry (STAR) who had baseline ASPECTS ≤ 5, LVO, and underwent EVT, there was no difference in the rates of symptomatic intracerebral hemorrhage (sICH) between patients who received IV tPA and those who did not (13.1% vs. 16.9%, *p* = 0.306) [16]. In another propensity-matched analysis of 282 patients with acute ischemic stroke enrolled in the International Stroke Perfusion Imaging Registry (INSPIRE) who achieved complete reperfusion after IV tPA or thrombectomy, there was increased sICH in the IV-tPA-only group with an infarct core >30 mL compared to those with EVT (20% vs. 3%, *p* = 0.008); of note, the majority of the EVT group also received IV tPA [17].

## 4. Endovascular Thrombectomy for Large-Core Ischemic Stroke

Patients presenting with established LCI represent a management challenge. Despite the potential for improvement in disability, serious concerns exist regarding the futility of recanalization when there is no salvageable brain tissue and there is risk of reperfusion injury, which can lead to sICH and potentially worsened outcomes [18]. This is especially true in patients who present in the late window of acute ischemic stroke.

In 2015, five pivotal trials demonstrated efficacy of EVT for patients presenting with anterior-circulation LVO (MR CLEAN, ESCAPE, REVASCAT, SWIFT PRIME, and EXTEND IA) [19,20,21,22,23]. In all of these trials, there was planned exclusion or unplanned under-representation of patients presenting with established LCI on initial imaging, with volume assessment most often relying on non-perfusion imaging or ASPECTS. For example, in MR-CLEAN, the trial allowed inclusion of patients with low ASPECTS; however, only 28 patients had a baseline ASPECTS 0–4, and the subgroup analysis was underpowered to detect significant difference in outcome (adjusted common OR, 1.09 [95% CI, 0.14 to 8.46]) [19]. In ESCAPE, patients with initial ASPECTS < 6 were excluded [20]. In REVASCAT, patients with ASPECTS < 7 on initial CT or <6 on initial DWI-MRI were excluded [21]. In SWIFT-PRIME, patients were excluded if there was involvement of >1/3 of the MCA territory on initial CT or MRI, if ASPECTS < 6 or if infarct volume in other territories was >100 cc [22]. In ESCAPE-IA, patients with ischemic core > 70 mL on CTP were excluded.

In the subsequent late-window EVT trials, patients with LCI continued to be excluded from enrollment. The median infarct volume on presentation was 7.6 mL (interquartile range [IQR] 12–18) in the intervention group of DAWN [24] (measured on DWI-MRI; infarcts > 50 mL were excluded), and 9.4 mL (IQR 2.3–25.6) in the intervention arm of DEFUSE 3 [25] (volume measured on CTP or DWI-MRI; infarcts > 70 mL were excluded).

The rationale for exclusion of patients with LCI from EVT trials was subsequently challenged by several observational studies showing the potential benefit and low risk of harm in carefully selected patients. In a pre-specified subgroup analysis of SELECT [1], a multicenter, observational prospective study of imaging modalities used to select for anterior circulation EVT, of 105 patients with large ischemic core (defined as ASPECTS of ≤5 on non-contrast CT or volume of ≥50 mL), 31% of those who were treated with EVT achieved functional independence (90-day mRS 0–2) compared to 14% of those treated with medical management only (OR, 3.27 [95% CI, 1.11–9.62]; *p* = 0.03). In addition, EVT was associated with less infarct growth (44 vs. 98 mL; *p* = 0.006) and smaller final infarct volume (97 vs. 190 mL; *p* = 0.001) compared to medical management alone. Notably, every hour of treatment delay was associated with a 40% reduction in the odds of functional independence. There was a numerically higher but not statistically significantly different rate of sICH with EVT (13% vs. 7% *p* = 0.51), but no difference in neurological worsening at 24 h or mortality at 90 days. As expected, patients with larger initial infarct volumes had lower odds of functional independence; the odds of a good outcome declined by 42% for each 10 mL increase in stroke volume on CTP. Only 10 patients with EVT had infarct volumes >100 mL, none of whom achieved good functional outcomes [1].

Subsequently, three randomized control trials evaluated EVT for LCI (Table 1). A trial conducted in Japan was among the first to enroll patients with LCI [26]. In the RESCUE-Japan LIMIT trial, Yoshimura et al. randomized 203 patients presenting with LCI (defined as ASPECTS 3–5 on CT or DWI-MRI) within 6 h of stroke onset; 101 received EVT plus medical care and 102 received medical care alone. EVT resulted in more than double the odds of good functional outcome, defined as mRS 0–3 (31% vs. 12.7%). The trial also showed similar rates of sICH within 48 h but higher rates of any intracranial hemorrhage in the EVT group. There were no differences in the rates of DHC or death. However, the generalizability of these results was subsequently challenged due to the inclusion of exclusively Japanese patients and the use of a lower dose of alteplase (0.6 mg/kg) prior to thrombectomy [27].

Subsequently, the SELECT-2 trial was designed and recently completed [28]. SELECT-2 was a randomized, open-label international clinical trial that randomized patients with LCI (defined as ASPECTS 3-5 on non-contrast CT or ≥50 mL on CTP or DWI-MRI) to EVT plus medical care vs. medical care alone. The trial was stopped early after enrolling 352 patients (178 to EVT plus medical care and 174 to medical care only) due to efficacy. Patients in the EVT arm were significantly more likely to shift their 90-day mRS towards better outcomes and had significantly higher rates of functional independence (relative risk 2.97); the rates of sICH in the two groups were very low and there was no difference in mortality. The outcome benefit persisted in subgroup analyses even in patients with larger ischemic core (including >100 mL) and those with lower ratios of mismatch between the ischemic penumbra and ischemic core.

At the same time as the SELECT-2 trial was published, the ANGEL-ASPECT trial was completed in China [29], which randomized 456 patients presenting with LCI > 6 h from onset (defined as ASPECTS 3-5 on non-contrast CT or infarct volume 70–100 mL on DWI-MRI). The trial was stopped early due to efficacy; EVT resulted in a significant shift of the mRS towards better outcomes. The rate of any ICH was significantly higher in the EVT group (49.1% vs. 17.3%); however, there was no significant difference in the rate of sICH. The rate of sICH was numerically higher for the EVT group (6.1% vs. 2.07%). There were no significant differences in rates of hemicraniectomy or death.

Despite differences in design, patient population, and reported rates of ICH and sICH between the three trials (likely owing to differences in definition of sICH), the results of these trials provide convincing evidence of the efficacy of EVT for LCI across different patient populations and imaging characteristics (Table 1).

It is important to note that despite the proven efficacy of EVT in LCI, the proportion of EVT patients who achieve functional independence is lower compared to those with small to medium infarcts. In a meta-analysis of individual patient data from MR CLEAN, ESCAPE, REVASCAT, SWIFT PRIME, and EXTEND IA (which excluded LCI) [30], the proportion of patients with mRS 0–2 (functional independence) at 90 days was 46%, compared to 20.3% in SELECT2, 30% in ANGEL-ASPECT, and 14% in RESCUE-Japan LIMIT, indicating a shift towards mRS 3 (moderate disability but able to walk unassisted) and 4 (moderately severe disability). Therefore, addressing expectations with patients undergoing EVT for LCI is essential. Although the magnitude of benefit observed in these cases might be comparatively weaker than that seen in patients with small to medium-sized infarcts, it is important to emphasize that the benefits likely remain clinically meaningful. Moreover, it is important to consider pre-stroke disability in deciding whether LCI patients are eligible for EVT; all three LCI EVT trials restricted enrollment to patients with pre-stroke mRS 0–1. Therefore, the outcome of EVT in LCI patients with high pre-stroke mRS is likely to be significantly worse than those without pre-stroke disability; however, this needs further research.

## 5. Neurocritical Management of Large-Core Ischemic Strokes

Patients with LCI require close monitoring of the neurological exam, as well as blood pressure, serum glucose, temperature, and respiratory and cardiac function. This is especially true after tPA or EVT, in order to ensure early detection of immediate complications such as hemorrhagic transformation, malignant cerebral edema, cerebral herniation, seizures, respiratory failure, and others. This optimally occurs in a specialized acute stroke unit or neurocritical care unit where such close monitoring is feasible.

### 5.1. Blood Pressure

Under normal circumstances, the resistance imparted by cerebral arterioles is the main determinant of local cerebral blood flow. In a process known as cerebral autoregulation, these arterioles modulate their smooth muscle tone in response to upstream blood pressure and local metabolic rate [31]. Ischemia leads to appropriate dilatation of cerebral arterioles. At the point of maximal arteriolar dilation, cerebral blood flow becomes heavily reliant on systemic arterial blood pressure, such that a minimal reduction in cerebral perfusion pressure can accelerate neuronal loss. This concept forms the basis for the approach of permissive hypertension in patients presenting with acute ischemic stroke before recanalization. With prolonged or severe ischemia, the arterioles are injured, which results in impairment of autoregulation. If recanalization occurs, this may increase the risk of reperfusion injury.

Several prior studies have suggested a U- or J-shaped relationship between blood pressure and clinical outcomes after ischemic stroke, indicating that both high and low blood pressures after stroke are associated with worsened outcomes. In an analysis of >17,000 patients enrolled in the International Stroke Trial (IST) with confirmed ischemic strokes, early death increased by 17.9% for every 10 mm Hg below a systolic blood pressure (SBP) of 150 mm Hg and by 3.8% for every 10 mm Hg above 150 mm Hg [32]. Although other studies have demonstrated similar findings [33], these studies did not evaluate the impact of LVO and revascularization status on the relationship between blood pressure and clinical outcomes. In a large retrospective study of 674 consecutive patients with acute stroke treated with intravenous thrombolysis or intra-arterial therapies [34], Martins et al. showed that the relationship between blood pressure within 24 h and the 3-month clinical outcome differed according to recanalization status. There was a J-shaped curve among patients with non-recanalized vessels, and a linear association between blood pressure and outcome among patients with recanalized vessels, suggesting that recanalization modulates the association between blood pressure and outcomes after LVO recanalization.

Therefore, it is logical to conclude that the degree of recanalization matters when deciding optimal blood pressure goals after recanalization. However, the exact blood pressure goal remains much debated. Evidently, blood pressure has been shown to spontaneously decrease more significantly and earlier in patients with successful recanalization than in those with unsuccessful recanalization [35]. In a post-hoc analysis of 3631 stroke patients treated with EVT and enrolled in the SITS-TBYR registry [36], higher SBP in the first 24 h in patients with successful recanalization (mTICI 2b-3) was independently associated with less functional independence (defined as mRS 0–2 at 3 months), more sICH, and mortality. In patients with unsuccessful recanalization (mTICI 0–2a), higher SBP was associated with more sICH but not with higher rates of functional independence. Among patients with successful recanalization, SBP of 100 to 119 mm Hg was associated with the highest rates of functional independence (63%). Interestingly, and contrary to the study by Martins et al. [34], the relationship between blood pressure and outcome was found to be non-linear among patients with successful recanalization. As blood pressure decreased, there was an increased likelihood of a favorable outcome; however, beyond a certain point, the trend reversed, and the chance of a favorable outcome decreased. The reason that some studies show a linear relationship while others show a J-shaped relationship between blood pressure and outcome may be due to differences in patient characteristics related to baseline blood pressure, the actual achieved blood pressure after EVT (i.e., some studies might lack patients at the extreme ends of blood pressures), and stroke etiology (i.e., patients with cardioembolic strokes may tolerate lower pressures than those with intracranial atherosclerosis, which is associated with impaired autoregulation) [37].

A post-hoc analysis of eight MR-CLEAN registry sites showed that higher maximum systolic pressures within 6 h following EVT was associated with worse functional outcome (adjusted common OR per 10 mm Hg increment, 0.93, [95% CI 0.88–0.98]) and a higher rate of sICH (adjusted OR, 1.17, [95% CI 1.02–1.36]). Importantly, the association between minimum SBP and functional outcome was non-linear with an inflection point at 124 mm Hg; rates of minimum SBP lower and higher than the inflection point were associated with worse functional outcomes [38]. While several studies confirmed the concept that higher post-EVT blood pressures are associated with worsened outcomes, others challenged it. The recent BP-TARGET trial showed that intensive SBP lowering to a range of 100–129 mm Hg within 1 h post-EVT did not result in a significant reduction in sICH at 24–36 h compared to a standard SBP target of 130–185 mm Hg; however, the study was not sufficiently powered to detect differences in functional outcome (Table 2) [39]. More recently, ENCHANTED 2/MT in China was the first clinical trial powered to detect differences in functional outcomes after EVT in relationship to blood pressure [40]. The trial randomized 821 patients with persistently elevated SBP (≥140 mm Hg for >10 min) following successful reperfusion with EVT (defined as eTICI 2b-3) to either more intensive blood pressure control (target SBP < 120 mm hg) or liberal blood pressure control (target 140–180 mm Hg). The mean achieved SBP was 125 mm Hg at 1 h and 121 mm Hg at 24 h in the more intensive group, versus 143 mm Hg at 1 h and 139 mm Hg at 24 h in the less intensive group. The study was stopped early due to efficacy and safety concerns; patients in the more intensive treatment group had worse scores on the mRS at 3 months than those in the less intensive group, along with higher rates of death or neurological deterioration at 7 days, major disability, and worse health-related quality of life at 3 months. There was no difference in sICH or all-cause mortality. Of note, the study population had high baseline rates of hypertension (>60%) and intracranial atherosclerosis (>40%), both of which may affect the generalizability of the results.

More recently, two RCTs demonstrated the lack of benefit in intensive BP lowering after successful EVT and the possibility of harm. BEST-II was an open-label, phase 2, futility-design RCT which aimed to determine whether lower SBP targets after successful EVT (with goals of <140 mm Hg or <160 mm Hg) compared with a higher target of ≤180 mm Hg met pre-specified criteria for futility [41]. Primary outcomes included infarct volume at 36 h and utility-weighted mRS at 90 days. The final infarct volumes suggested a trend toward benefit in the lower SBP target group; however, this may have been mediated by a lower baseline infarct volume in the group targeting SBP < 140 mm Hg. The point estimate of treatment effect on the utility-weighted mRS was in the direction of harm, with a 0.0019 reduction in the utility-weighted mRS score for each mm Hg reduction in the SBP target; *p*-value for futility was 0.93. Although the criteria for futility were not met, the trial suggested a low probability of benefit from lower SBP targets after endovascular therapy if tested in a future larger trial. Published at the same time as BEST-II, OPTIMAL-BP was an open-label RCT conducted across 19 stroke centers in South Korea that randomized patients with successful thrombectomy (TICI2b or higher) with SBP > 140 after EVT to intensive BP control (SBP < 140) or conventional BP control (SBP 140–180) for 24 h after enrollment [42]. The trial was terminated early due to safety concerns during interim analysis; patients in the intensive group were less likely to achieve functional independence (mRS 0–2) at 3 months, and there were no differences in the incidence of sICH, stroke-related mortality, infarct volume or quality of life measures at 3 months. Those in the intensive group also had increased risk of malignant cerebral edema. The trial had several limitations; the rate of time spent in the target SBP range (140–180 mm Hg) was only 42.1% in the conventional management group because of spontaneous BP decline below 140 mm Hg, which may under-power the trial. Additionally, the mRS primary end point was ascertained using a patient-reported rather than clinician-rated algorithm.

While the optimal blood pressure target post-EVT remains uncertain, the existing data indicate that extremes of SBP should be avoided, specifically SBP < 120–130 and >180. A more individualized approach, taking into account baseline blood pressure, presence of intracranial atherosclerosis, final infarct volume and medical comorbidities, may be needed.

Unfortunately, the majority of the studies discussed above did not analyze the effect of the final infarct volume on blood pressure goal after EVT. In patients with LCI without EVT or tPA, acute blood pressure lowering is typically avoided for the first few days unless it is greatly elevated (>220/120), while patients who receive tPA are maintained below 180/105 for the first 24 h [43]. Achieving these targets typically requires a temporary discontinuation of home antihypertensive regimens, followed by their re-initiation 24–48 h after stroke onset. Patients with certain comorbid conditions such as myocardial infarction, heart failure, and aortic dissection will require lower blood pressure targets. Therefore, deciding on such targets necessitates a balanced risk–benefit analysis to assess the potential risks and benefits associated with lowering blood pressure. The optimal blood pressure target for patients who go on to develop a large infarct despite successful recanalization is still not well-established. Such patients might be at a higher risk of reperfusion injury and sICH compared to non-recanalized patients due to the combination of infarct volume and open vessels. Further research is needed to determine the most appropriate blood pressure management strategy in this specific group of patients. Irrespective of the blood pressure target, it is important to minimize blood pressure variability given its consistent association with worsened outcomes after stroke [44]. Calcium channel blockers, such as intravenous nicardipine or clevidipine, are widely used to treat high blood pressure as they are associated with reduced BP variability compared to other agents [45].

Hypotension is uncommon at the time of stroke and typically suggests the presence of an underlying medical condition. It is crucial to avoid hypotension and promptly correct it, as low blood pressure is also linked to an unfavorable outcome [32,38]. On occasion, augmenting blood pressure in carefully selected patients with vessel occlusions and otherwise normal blood pressure is attempted in order to enhance cerebral perfusion through collateral circulation or a partially occluded vessel; however, this practice is controversial, it has not been proven in clinical trials, it may be associated with increased cerebral edema and myocardial injury, and there is limited data on the chronic effects of augmented perfusion [46]. Regenhardt et al. proposed an approach to induced hypertension that involves titrating BP to an improvement of the neurologic exam, or augmenting mean arterial pressure by 10–20% (using phenylephrine or norepinephrine), in patients with expected delay to EVT, unsuccessful recanalization, or large-vessel occlusion patients who are not candidates for EVT, especially those with a BP- or position-dependent exam, DWI-clinical mismatch, or DWI-PWI mismatch indicating the presence of a residual penumbral tissue at risk of further ischemic injury [46].

### 5.2. Hemorrhagic Transformation

Hemorrhagic transformation (HT) is one of the most feared complications of acute ischemic stroke due to the associated increased risk of morbidity and mortality, typically occurring within 2 weeks of stroke onset. Ischemic injury leads to disruption of the blood–brain barrier. HT is commonly classified radiographically into hemorrhagic infarction (HI) or parenchymal hematoma (PH) based on CT appearance, in a widely used classification system that was first introduced by the ECASS investigators [47]. HI is characterized by CT evidence of scattered distribution of areas of high attenuation (i.e., petechial hemorrhages) at the infarct margin (HI1) or throughout the infarct without mass effect (HI2). PH is defined as a homogeneous region of circumscribed high attenuation (i.e., parenchymal hematoma) involving either ≤ 30% if the infarcted area (PH1) or >30% with mass effect (PH2) [48]. In ECASS, only PH2 was associated with early neurological deterioration and 3-month mortality [49].

Due to concerns regarding inter-rater reliability, the absence of reporting on other types of HT (including subarachnoid hemorrhage, intraventricular hemorrhage, and remote parenchymal hemorrhage), the lack of clinical criteria, and the absence of patients who underwent EVT in the original ECASS trials, alternative classification systems have been introduced that aim to differentiate clinically significant HT from purely radiographic findings and also take into account the impact of EVT. One such classification system is the Heidelberg Bleeding Classification (Table 3) [50], in which sICH is defined as a new intracranial hemorrhage detected on brain imaging and associated with neurologic deterioration (defined as an increase ≥ 4 in the total NIHSS or ≥2 points in one NIHSS subcategory) [50].

The prevalence of HT in clinical studies varies depending on the definition and on whether tPA or EVT were used. In a systematic review of 65 stroke studies that used the ECASS-2 definitions of HT (n = 17,259), overall pooled HT prevalence was 27% (95% CI 23–30); 32% (95% CI 27%–37%) in those with tPA; and 20% (95% CI 14%–27%) in those without tPA [50]. However, the rate of clinically meaningful HT is significantly lower. In a meta-analysis of 9 randomized trials of tPA vs. placebo (n = 6756 patients) [51], the rate of sICH (defined as PH2 with a deterioration of ≥4 points on NIHSS) was 3.7% in those with tPA vs. 0.6% in those without tPA, and the rate of fatal ICH was 2.7% in those with tPA vs. 0.4% in those without tPA. In patients with LVO, EVT does not seem to affect the risk of clinically meaningful HT. In the meta-analysis of individual patient data from MR CLEAN, ESCAPE, REVASCAT, SWIFT PRIME, and EXTEND IA [30], the rates of sICH and PH2 were comparable between those who underwent EVT and those who did not (~4% for sICH and ~5% for PH2). However, these trials excluded LCI. In the clinical trials of EVT for LCI discussed above, there were numerical differences in rates of sICH between patient with and without EVT: 13% vs. 7% in SELECT [1], 9% vs. 4.9% in RESCUE-Japan LIMIT [26], 6.1% vs. 2.1% in ANGEL-ASPECT [29], and 1.2% vs. 1.1% in SELECT-2 [28]. None of these differences were statistically significant. The difference in the rates of sICH among these trials is likely due to heterogeneity of patient populations and variable definitions of sICH.

Risk factors for post-tPA sICH include older age, greater stroke severity, hyperglycemia, hypertension, heart failure, kidney disease, atrial fibrillation, use of antiplatelet and anticoagulant agents, and white matter changes on brain imaging, among others [52]. While there are several sICH prediction scores available, they should not be utilized to justify withholding thrombolytic therapy [53]. It is important to consider that patients with the highest predicted risk of sICH are also the most likely to have poor outcomes if thrombolytic therapy is not administered.

Close neurologic monitoring and early diagnosis of HT is important to prevent further complications. Therefore, serial neurological examinations in a neurocritical care unit or acute stroke unit are recommended, and emergent brain imaging (CT) in the event of new headaches, nausea, vomiting, or neurological deterioration, especially in the first 24 h after tPA, is critical for early recognition [43,53,54]. When symptomatic HT occurs within 24 h of tPA (or in the setting of hypofibrinogenemia), reversal of tPA is warranted. As soon as sICH is identified, serum fibrinogen level should be checked, followed by empiric administration of 10 units of cryoprecipitate. Additional units of cryoprecipitate should be given to maintain fibrinogen levels > 150 mg/dL. The anti-fibrinolytics tranexamic acid or aminocaproic acid can be administered if there is a contraindication to cryoprecipitate or a delay in obtaining it. Additional agents can be considered based on the clinical scenario and other associated coagulopathies, such as platelet transfusion in patients with platelet count < 100,000/µL, prothrombin complex concentrate (PCC), fresh-frozen plasma (FFP) and/or Vitamin K in patients with recent warfarin treatment or elevated INR, and aminocaproic acid or tranexamic acid in patients who decline blood products [53]. Additionally, blood pressure control is often employed to reduce the risk of further hematoma expansion. However, it is important to weigh the benefits of lowering blood pressure to prevent hematoma expansion against the risk of worsening ischemia in patients with LCI. The optimal blood pressure target after HT is unclear and may need to be individualized based on the extent of hemorrhage and whether recanalization with thrombolysis or EVT has been achieved. Despite the lack of data, patients with smaller HT (HI1 or 2) and incomplete or unsuccessful recanalization may benefit from a more liberal blood pressure strategy (SBP < 160 or <180), while patients with hematomas (PH1 or 2) and complete recanalization may be at higher risk of hematoma expansion and more likely to benefit from more intensive blood pressure control (SBP < 140). In the case of rapidly expanding hematomas, herniation, or malignant edema, DHC with or without hematoma evacuation may be needed [55].

### 5.3. Malignant Cerebral Edema

#### 5.3.1. Diagnosis and Monitoring

Malignant cerebral edema (MCE) is characterized by accumulation of cytotoxic edema which leads to progressive clinical deterioration. Without treatment, it imparts a very high risk of herniation and death. Most often, this develops after large infarcts in the MCA territory (equivalent to ≥1/2–2/3). Infarctions from MCA branch occlusions typically do not result in MCE or clinically significant mass effect necessitating DHC [56].

By definition, any patient with an LCI might be at risk for MCE, which occurs in 10–78% of patients with MCA infarction [57], but could also occur in other territorial infarcts [58]. Established risk factors for MCE include younger age, larger infarct volume (≥1/2 MCA territory), higher admission NIHSS, poor collateral flow, midline shift >3.7–5 mm in the first 24–48 h and unsuccessful recanalization with tPA or EVT [11,59,60,61]. Patients at risk for MCE are typically monitored in a neurocritical care or the acute stroke unit where close neurologic (every 1 or 2 h), cardiac and respiratory monitoring is feasible. Intubation and mechanical ventilation is often necessary to secure the airway and prevent aspiration. The progression of cerebral edema and mass effect typically peaks in the first 3–5 days and, if left untreated, results in progressive midline shift, transtentorial herniation, compression of thalamus and brainstem and subsequently brain death [56]. A subset of patients develop neurological deterioration later than this “swell window” due to progressive infarction of penumbral tissue and/or HT. A decline in the level of consciousness (LOC) is often the first sign of MCE, which can be challenging to clinically follow in patients with eyelid opening apraxia or concomitant ACA infarctions (which could affect LOC) [58]. In patients where neurological examination is not reliable, serial CT scans are necessary. Pupillary abnormalities are an indication of life-threating MCE resulting in herniation and midbrain compression and should prompt evaluation and management. Despite the massive localized mass effect, routine ICP monitoring is not indicated for several reasons [58]; most patients with large hemispheric infarcts have normal ICP [62], CT changes do not always correlate with ICP, and patients with MCE typically do not have significant ICP elevations until after clinical herniation [63].

#### 5.3.2. Management of MCE

The management of MCE is tailored towards the prevention of secondary brain injury due to tissue displacement from mass effect. Several medical interventions are often attempted in a deteriorating patient with MCE; however, none have been systematically shown to improve neurological outcomes. These interventions include general ICP-lowering measures, such as head of bed elevation, transient hyperventilation, cerebrospinal fluid diversion, osmotic therapy, and sedation [64]. In patients with rapid neurological deterioration, without surgical intervention, 40–80% of patients with MCE die [65,66], and those who survive develop severe disability. Therefore, neurosurgical consultation should be obtained in all patients at risk for MCE; medical interventions are used only as a bridge to definitive surgical treatment.

Hyperosmolar therapy is the mainstay of medical therapy in patients with cerebral edema, including those with MCE [64]. Mannitol and hypertonic saline are the most commonly used agents, which exert their effect by creating an osmotic gradient to move water from the cerebral interstitial tissue to the cerebral vasculature. This requires an intact blood–brain barrier (BBB), and thus hyperosmolar therapy predominantly reduces the volume of normal, uninfarcted tissue [67,68]. Although both are effective at acutely reducing ICP, neither has been shown to improve functional outcomes or mortality after stroke [69,70,71,72]. Consequently, 20–25% mannitol (0.25–2 g/kg as a bolus infusion over 10 min) and 23.4% hypertonic saline (30 mL over 2–5 min) are frequently given to rapidly deteriorating patients with MCE as preparations for DHC are underway. Repeated boluses of either mannitol and hyperosmolar therapy should not be utilized prophylactically or to delay or circumvent DHC [64].

Several randomized clinical trials have evaluated the efficacy of DHC compared to maximal medical therapy in patients with MCE or large, space-occupying hemispheric infarction [2]. These include DECIMAL (2007) [73], DESTINY (2007) [74], HAMLET (2009) [75], Slezins et al. (2012) [76], Zhao et al. (2012) [77], HeADDFIRST (2014) [65], DESTINY II (2014) [78], and HeMMI (2015) [79]. These trials varied in their inclusion criteria regarding age and baseline functional status, infarct size, time to surgery, definition of standard medical management, and primary outcomes. They were also limited by small sample size, absence of blinding, exclusion of older patients, and lack of reporting regarding withdrawal of life-sustaining treatment and mortality outcomes. Despite these limitations, these trials established the benefit of early DHC on mortality and improving functional outcomes. In a pooled analysis of the three original trials (DESTINY, DECIMAL, and HAMLET) including patients ≤ 60 years old and treated within 48 h, the number needed to treat (NNT) was 4 to achieve a mRS ≤ 3 (absolute risk reduction [ARR] = 23%), 2 to achieve a mRS ≤ 4 (ARR = 51%), and 2 to survive irrespective of mRS (ARR = 50%) [80]. A subsequent pooled analysis of all eight trials [2] showed substantial improvement in the chance of favorable outcomes (mRS ≤ 3) at 1 year, even after adjustment for age, sex, baseline stroke severity (NIHSS), presence of aphasia, and time from stroke onset to randomization (adjusted OR, 2.95 [95% CI 1.55–5.60], *p* = 0.001) and significant reduction in mortality (1 year adjusted OR 0.16 [95% CI 0.10–0.24], *p* < 0.001). Among patients 60–82 years old, there remains a clear survival benefit, but the majority of survivors achieve a mRS of 4 or 5 [81]. The optimal timing of DHC within the first 48 h is based off the RCTs discussed above. Only HeADDFIRST, HeMMI and HAMLET allowed randomization after 48 h; a subgroup analysis in HAMLET found no benefit of DHC on neurological outcomes or mortality when performed after 48 h [75]. In an analysis of 1301 patients undergoing DHC extracted from the Nationwide Inpatient Sample between 2002 and 2011 [82], 55.8% underwent surgery within 48 h. When time was assessed as a continuous variable, later surgery was associated with increased odds of discharge to institutional care and poor outcome. When evaluated dichotomously, the odds of discharge to institutional care and poor outcome did not differ at 48 h after hospital admission, but increased when surgery was performed later than 72 h. Importantly, there was no association between surgical time and outcomes prior to herniation, indicating that DHC should be performed prior to clinical herniation. Thus, waiting for neurological deterioration is not advised. The benefit of performing DHC within 24 vs. 48 h remains uncertain [83,84,85]. Contrary to the commonly held perception that global aphasia leads to poorer functional outcomes and reduced quality of life, there is insufficient substantial evidence to justify withholding DHC from patients with dominant hemispheric infarcts. This is underscored by the possibility of certain patients experiencing significant recovery in language function, extending up to 25 years after stroke [86].

Surgical decompression is typically performed through a fronto-temporo-parietal craniectomy with a minimum anteroposterior length of 12 cm and a minimum superoinferior length of 9 cm [87,88,89]. After surgery is performed, patients should be closely monitored for complications of DHC including bleeding, infection (surgical wound infection or meningitis), CSF leak, subdural hygroma, seizure, sunken-flap syndrome, paradoxical herniation, and brain injury from external trauma in the presence of a skull defect. Despite limited evidence, prophylactic anti-seizure medications (ASM), most commonly levetiracetam, are commonly prescribed for the first 3–7 days after surgery to prevent early seizures. However, they are unlikely to affect long-term risk of late-onset seizures or post-stroke epilepsy [90,91,92]. A protective helmet is typically worn by patients after DHC when they are out of bed or working with physical therapy to protect the unshielded brain from external trauma. Cranioplasty, reconstruction of the skullcap removed during DHC, is typically performed 2–6 months after DHC [93].

#### 5.3.3. Unproven and Emerging Therapies for MCE

Over 1000 experimental therapies have been investigated for neuroprotective properties following ischemic stroke, both in preclinical and clinical studies, with no evidence of meaningful benefits in humans [94]. In the context of MCE due to LCI, corticosteroids have been examined with no conclusive benefit on mortality or functional outcomes [95]. Barbiturates might be effective at reducing ICP through lowering metabolic demand; however, they are associated with serious adverse effects and have not been shown to improve outcomes [96]. Temperature control has been of interest for decades; several early observational studies and small randomized trials described potential beneficial effects of therapeutic hypothermia as a neuroprotective measure in MCE with or without DHC [97,98,99,100]; however, recent data have shown potential for harm. Neugebauer et al. [101] randomized 50 patients aged 18–60 years with MCE who were treated with DHC within 48 h to receive moderate hypothermia within 12 h of DHC (33.0 ± 1.0 °C, maintained for 72 h) or standard of care. The trial was stopped early (short of the projected plan to include 324 patients) due to safety concerns; 12 of 26 patients (46%) in the hypothermia group and 7 of 24 patients (29%) receiving standard care had at least one serious adverse event within 14 days (OR, 2.05 [95% CI, 0.56–8.00]; *p* = 0.26); after 12 months, rates of serious adverse events were 80% in the hypothermia group and 43% in the standard care group (hazard ratio, 2.54 [95% CI, 1.29–5.00]; *p* = 0.005). There was no difference in 14-day mortality or 12-month functional outcome between the two groups. Whether there is a subset of patients who may benefit from therapeutic hypothermia, such as those who are not candidates for DHC, remains uncertain [98]. Trials of intravascular hypothermia in patients receiving EVT are underway [102,103].

Importantly, fevers should still be aggressively treated, as they have been consistently associated with poor outcomes [104,105]. Acetaminophen and cooling devices may be necessary to ensure adequate temperature control in patients with LCI who develop fevers while infectious workup is underway. Hyperglycemia is also common in patients with LCI [106], and is associated with increased mortality [107], increased rates of hemorrhagic events, and worse functional outcomes [108].

One of the most promising therapeutic targets for cerebral edema is the sulfonylurea receptor 1-immediate receptor potential melatonin 4 (SUR1-TRPM4) channel, which is upregulated in response to ischemic injury and contributes to cytotoxic edema [109]. Inhibition of SUR1-TRPM4 decreases infarct volume and mitigates edema in animal models of ischemic stroke [110,111]. IV glyburide (RP-1127; glibenclamide), an inhibitor of SUR1-TRPM4, is a promising therapy for MCE [112,113,114]. The safety and efficacy of intravenous glyburide on brain swelling after large hemispheric infarction (GAMES-RP) trial was a phase-2 trial that randomized 86 adult patients ≤80 years old with a diagnosis of anterior-circulation ischemic infarct (infarct volume 82–300 mL on DWI-MRI) to IV glyburide or placebo within 10 h of stroke onset. IV glyburide was tolerated without significant differences in adverse events. There was no difference in the rate of good functional outcome at 90 days (mRS 0–4) between the two groups (adjusted OR 0.87 [95% CI 0·32–2·32], *p* = 0.77); however, the study was stopped early due to funding reasons, limiting the ability to draw definitive conclusions about efficacy [113]. In a secondary analysis of GAMES-RP, IV glyburide was associated with improvements in midline shift, level of alertness, and NIHSS, and there were fewer deaths attributed to edema in the treatment group [112]. In another exploratory analysis evaluating long-term outcomes in patients enrolled in the GAMES-PR trial, patients ≤ 70 years old had improved survival at 12 months with IV glyburide [115]. The Efficacy and Safety of Intravenous BIIB093 (Glibenclamide) for Severe Cerebral Edema Following Large Hemispheric Infarction (CHARM) trial is currently underway [116].

Other experimental therapies for cerebral edema require further study; examples include other SUR1 inhibitors (such as glimepiride and resveratrol), vascular-endothelial growth factor (VEGF) inhibitors, aquaporin-4 (AQP4) inhibitors, microRNAs, and other BBB protective agents [117].

## 6. Seizures

Stroke is the most common cause of seizures and epilepsy in older adults, accounting for approximately one-third of new-onset seizures and epilepsy in individuals ≥ 65 years old [118]. Post-stroke seizures can be classified into two categories: early seizures (or acute symptomatic seizures), occurring within the initial week after stroke, and late seizures (or remote symptomatic seizures), which manifest after the first week. Notably, the risk of seizures persists beyond the acute phase, progressively increasing and often leading to the development of epilepsy months to years following the stroke [119,120]. The emergence of early post-stroke seizures can be attributed to various factors, including neuronal damage resulting from hypoxia, metabolic dysfunction, reperfusion injury, glutamate excitotoxicity, and disruption of the BBB. The onset of late seizures and post-stroke epilepsy is associated with gliotic scarring, chronic inflammation, altered synaptic plasticity, and other neurodegenerative processes that collectively contribute to the epileptogenic process [121]. It is important to note that a single, unprovoked, remote symptomatic post-stroke seizure qualifies as structural epilepsy due to the high risk or recurrence (>60%) within the next 10 years [122].

Risk factors for post-stroke seizures include younger age, increased stroke severity, cortical involvement, and anterior circulation infarcts [120]. Patients with LCI are at an especially high risk of both early- and late-onset seizures [123]. There is conflicting data on whether reperfusion therapies increase the risk of post-stroke seizures [124,125,126]. The routine prophylactic administration of ASMs in the primary prevention of post-stroke epilepsy is not recommended due to the lack of evidence regarding efficacy and potential for harm. Evidence suggests that some ASMs (especially phenytoin and benzodiazepines) may hamper mechanisms of neural plasticity that are essential to recovery after stroke [127,128,129,130]. It is worth noting that the absence of beneficial effects observed in previous studies for primary prevention might be attributed to the use of older-generation ASMs. Whether newer ASMs, such as levetiracetam and lacosamide, may decrease risk of post-stroke seizure and epilepsy needs further study. Short-term secondary prophylaxis is needed for patients who develop post-stroke seizures during hospitalization, with close follow-up needed to ensure discontinuation of ASM after the acute hospital setting [131]. Individual patient risk factors and preferences related to ASM side effects should be taken into consideration when choosing ASMs for secondary prophylaxis.

## 7. Anticoagulation Initiation in Large-Core Infarcts

The need to determine optimal timing for starting anticoagulant (AC) therapy in patients with LCI often arises when there is a clinical indication for early initiation or re-initiation of AC, such as atrial fibrillation, mechanical heart valve, cardiac thrombus, deep vein thrombosis, or pulmonary embolism. The risk of symptomatic HT with anticoagulation versus the risk of recurrent ischemic stroke or other systemic thromboembolism without AC must be carefully weighed [132]. Predictors for HT include large volume infarct, previous intracranial hemorrhage, thrombocytopenia, mechanical thrombectomy, and cerebral microhemorrhages [133]. The choice of AC depends on the indication for AC and patient-specific comorbidities; however, direct oral anticoagulants (DOACs), including apixaban, rivaroxaban, edoxaban, and dabigatran, are preferred in most clinical settings due to the reduced risk of intracerebral hemorrhage [134]. However, in patients with valvular atrial fibrillation, cardiac thrombus, or thrombotic antiphospholipid syndrome, warfarin is recommended over DOAC [135,136,137,138].

The timing of AC initiation has been extensively studied. In a meta-analysis (2007) of seven trials (4624 patients) comparing IV unfractionated heparin or low-molecular-weight heparin (LMWH) initiated within 48 h of a cardioembolic stroke versus other treatments (aspirin or placebo), IV anticoagulation was associated with an increase in symptomatic intracerebral hemorrhage (2.5% vs. 0.7%, OR 2.89 [95% CI: 1.19–7.01]), without statistically significant reduction in recurrent ischemic stroke within 7 to 14 days (3.0 vs. 4.9%, OR 0.68 [95% CI: 0.44–1.06]) [139]. The Early Recurrence and Cerebral Bleeding in Patient with Acute Stroke and Atrial Fibrillation (RAF) study showed that in patients with acute ischemic stroke and atrial fibrillation, a high CHA2DS2-VASc score, high NIHSS, large ischemic lesions, and choice of oral anticoagulant (OAC) independently led to greater risk of both recurrent ischemic stroke and major bleeding at 90 days; the best time for initiation of OAC was between 4 and 14 days from ischemic stroke (Table 4) [140]. Subsequently, the American Heart Association/American Stroke Association guidelines (2018) recommend starting OAC within 4 to 14 days after an acute ischemic stroke for most patients, with a further delay for patients with HT [141]. On the other hand, the European Society of Cardiology and European Heart Rhythm Association guidelines (2016) recommend the use of the “1-3-6-12-day rule”, which calls for initiation of OAC 1 day after a transient ischemic attack, 3 days after stroke with NIHSS < 8, 6 days after stroke with NIHSS 8 to 15, and 12 days after stroke with NIHSS > 15 [142]. Since these guidelines, several observational studies and trials have shown that earlier OAC initiation is likely safe and associated with reduced ischemic events without an associated increase in HT (Table 4). Recently, initiation of DOAC within 1 day (for TIA), 2 days (for NIHSS < 8), 3 days (for NIHSS 8–15), or 4 days (for NIHSS ≥ 16), known as the “1-2-3-4 day rule”, was shown to decrease risk of recurrent ischemic stroke with no increase in major bleeding [143]. Subsequently, the Timing of Oral Anticoagulant Therapy in Acute Ischemic Stroke with Atrial Fibrillation (TIMING) trial (2022) showed that early initiation (≤4 days) of DOAC was non-inferior to delayed initiation (5–10 days) in relation to a composite of recurrent ischemic stroke, symptomatic intracerebral hemorrhage, or all-cause mortality at 90 days. Of note, the majority of patients in TIMING had mild to moderate-size strokes [144].

Most recently, the Early Versus Late Initiation of Direct Oral Anticoagulants in Post-Ischemic Stroke Patients with Atrial Fibrillation (ELAN) trial randomized patients with acute ischemic stroke and atrial fibrillation to either early (≤48 h after minor/moderate stroke, day 6–7 after major stroke) or later (day 3–4 after minor stroke, day 6–7 after moderate stroke, day 12–14 after major stroke) initiation of DOAC therapy. An infarct of 1.5 cm or smaller was defined as minor; an infarct in the distribution of a cortical superficial branch of the middle, anterior, or posterior cerebral artery was defined as moderate; and larger infarcts in the distribution of these arteries or a brainstem or cerebellar infarct larger than 1.5 cm were defined as major. Early versus later DOAC initiation resulted in similar rates of the composite primary outcome of recurrent ischemic stroke, systemic embolism, major extracranial bleeding, sICH, or vascular death (early vs. late 2.9% vs. 4.1%, OR 0.7 [95% CI 0.44 to 1.14]) [145]. The rates of all components of the primary outcome were low. Interestingly, even in the subgroup with large infarcts, there was no increased sICH risk with early AC. The evidence from this trial suggests that earlier AC after large stroke is safe. Given the exclusion or underrepresentation of patients with HT from most early AC trials, the timing of AC initiation for this population warrants further investigation [146].

## 8. Goals of Care

The prognosis and long-term outcomes of patients with LCI depend on various factors, including age, baseline functional status, the efficacy of reperfusion therapies, the success of DHC, development of HT, and other hospital-related complications. The majority of patients with LCI sustain at least mild to moderate disability, with high rates of depression, cognitive dysfunction and anxiety [147]; therefore, conversations about long-term outcomes are typically conducted with families to establish the patient’s personal goals and to set realistic expectations. It is recommended to adopt an individualized approach when engaging in discussions about care goals. Acceptable levels of disability to patients and families vary, necessitating an individualized evaluation of what constitutes a “favorable” or “unfavorable” outcome based on patient-specific objectives and acceptable functional levels.

Furthermore, addressing specific, predicted functional losses can assist families during this challenging period. For instance, while some patients might accept being wheelchair-bound but not entirely aphasic, others may find any level of disability unacceptable. Consequently, it is crucial to thoroughly discuss the stroke’s anatomic and functional impact, the potential need for procedures like tracheostomy or percutaneous gastrostomy, as well as the expected duration of rehabilitation with patients and their families. The premature withdrawal of life-sustaining therapies due to perceived “poor prognosis” should be avoided unless there is a clear indication that the patient previously expressed unwillingness to live with the expected extent of disability. The majority of stroke recovery occurs within the first 3 months [148], and patients continue to recover after 6–12 months, albeit at a much slower pace. Therefore, a more reliable prognosis can be determined 3–6 months after stroke onset, by which time some patients have the potential to recover up to 70% of their lost function [149,150].

## Figures and Tables

**Table 1:** Summary of trials evaluating endovascular treatment for large-core infarcts.

Trial (Year)	RESCUE-Japan LIMIT (2022) [26]	SELECT-2 (2023) [28]	ANGEL-ASPECT (2023) [29]
Study Design	Open-label, parallel-group RCT	Open-label, parallel-group adaptive RCT	Open-label, parallel-group RCT
Setting	Japan; 45 sites	Canada, United States, Europe, Australia, New Zealand; 31 sites	China; 46 sites
Dates Recruiting	November 2018–September 2021	September 2019–September 2022	October 2020–May 2022
Participants, n	203	352	456
Imaging Type	CT/MRI for ASPECTS CTA/MRA for site of occlusion	CT for ASPECTS CTP/MRP for infarct core volume	CT/MRI for ASPECTS CTP/MRP for infarct core volume CTA/MRA for site of occlusion
Imaging Criteria	ASPECTS score 3–5	ASPECTS score 3–5 ASPECTS > 5 if core > 50 mL	ASPECTS score 3–5 ASPECTS < 3 if core 70–100 mL ASPECTS > 5 if core 70–100 mL, >6 h from LKWT
Mismatch	Not required	Not required	Not required
Site of Occlusion	Terminal ICA, MCA M1	Cervical or intracranial ICA, MCA M1, tandem occlusion	Terminal ICA, MCA M1, tandem occlusion
Time Window	<6 h LKWT, or 6–24 h if FLAIR (-)	<24 h stroke onset	<24 h LKWT
Age (years)	>18	18–85	18–80
NIHSS	≥6	≥6	6–30
IV Thrombolysis	tPA (0.6 mg/kg)	tPA (0.9 mg/kg), TNK (0.25 mg/kg)	tPA (0.9 mg/kg), urokinase (1–1.5 million IU)
Exclusion Criteria	Mass effect with MLS Acute ICH Baseline coagulopathy (platelet count < 40 × 10^9^/L, INR > 3, aPTT > 50 s) Evidence of chronic occlusion	Mass effect with MLS Acute ICH Multiple occlusions (besides ICA, ipsilateral MCA M1)	Mass effect with MLS Acute bilateral strokes Multiple intracranial occlusions Baseline coagulopathy (platelet count < 100 × 10^9^/L, INR > 1.7, aPTT > 35 s)
Intervention	EVT with any device, angioplasty, stent	EVT with any locally approved device	EVT with devices approved by CFDA, IA thrombolysis, angioplasty, stent
Primary Outcome	mRS 0–3 at 90 days	Ordinal shift in mRS at 90 days	Ordinal shift in mRS at 90 days
Study Population			
Thrombolysis Administered	EVT 26.7% Medical Care 28.4%	EVT 20.8% Medical Care 17.3%	EVT 28.7% Medical Care 28.0%
Median Infarct Volume (IQR)—mL	EVT 94 (66–152) Medical Care 110 (74–140)	EVT 81.5 (57–118) Medical Care 79 (62–111)	EVT 60.5 (29–86) Medical Care 63 (31–86)
Median ASPECTS (IQR)	EVT 3 (3–4) Medical Care 4 (3–4)	ETV 4 (3–5) Medical Care 4 (4–5)	EVT 3 (3–4) Medical Care 3 (3–4)
Successful Reperfusion (≥ TICI 2b)	86.0%	79.8%	81%
Results			
Primary outcome	mRS 0–3 at 90 d 31% vs. 12.7% (EVT vs. medical therapy); RR 2.43 (95% CI 1.35–4.37), *p* = 0.002	Shift toward better outcomes in EVT group; OR 1.51 (95% CI 1.20–1.59), *p* < 0.001 Higher rate of functional independence (mRS 0–2) for EVT, 20% vs. 7%; RR 2.97 (95% CI 1.60–5.51)	Shift toward better outcomes on mRS in EVT group; OR 1.37 (95% CI 1.11–1.69), *p* = 0.004
Rate of Symptomatic ICH	No significant difference; 9.0% for EVT vs. 4.9% medical management, *p* = 0.25	No significant difference; 0.6% for EVT vs. 1.1%, RR 0.49 (95% CI 0.04–5.36)	No significant difference, 6.1% for EVT vs. 2.7% medical management (*p* = 0.12)
Rate of any ICH	Higher rate for EVT (58% vs. 32%, *p* <0.001)	No significant difference	Higher rate for EVT (49.1% vs. 17.3%, *p* <0.001)
Other Adverse Events	No significant difference in death or DHC	More vascular complications in EVT No significant difference in mortality	No significant difference in death or DHC
Limitations	Single country High rates of MRI to determine ASPECTS (88%) No use of perfusion imaging Standard tPA dose in Japan lower than that in many other countries	Early termination limits subgroup analysis Low percentage receiving thrombolysis	Single country Low percentage receiving thrombolysis No patients enrolled with ASPECTS > 5 and core 70–100 cc
Notes		Stopped early due to efficacy	Stopped early due to efficacy

Abbreviations: RCT, randomized controlled trial; aPTT, activated partial thromboplastin time; ASPECTS, Alberta Stroke Program Early CT Score; CFDA, China Food and Drug Administration; CI, confidence interval; CTA, computed tomography angiography; CTP, computed tomography perfusion; DHC, decompressive hemicraniectomy; EVT, endovascular treatment; IA, intra-arterial; ICA, internal carotid artery; ICH, intracranial hemorrhage; INR, international normalized ratio; LKWT, last known well time; M1, first segment of middle cerebral artery; MCA, middle cerebral artery; MLS, midline shift; MRA, magnetic resonance angiography; MRP, magnetic resonance perfusion; mRS, modified Rankin score; NIHSS, National Institutes of Health Stroke Scale; OR, odds ratio; sICH, symptomatic intracerebral hemorrhage; TICI, thrombolysis in cerebral infarction score; TNK, tenekteplase; tPA, tissue plasminogen activator.

**Table 2:** Summary of major trials evaluating blood pressure management after endovascular treatment of large-vessel occlusion stroke.

Trial (Year)	BP-TARGET (2021) [39]	ENCHANTED2/MT (2022) [40]	BEST-II (2023) [41]	OPTIMAL-BP (2023) [42]
Study Design	Open-label RCT	Open-label RCT	Open-label, futility-design RCT	Open-label RCT
Setting	France; 4 academic centers	China; 44 hospitals	USA; 3 stroke centers	South Korea: 19 stroke centers
Dates Recruiting	June 2017–September 2019	July 2020–March 2022	January 2020–March 2022	June 2020–November 2022
Participants, n	324	821	120	302
Inclusion Criteria	Age ≥ 18 Acute ischemic stroke due to LVO treated with EVT TICI 2b, 2c, or 3 reperfusion	Age ≥ 18 Acute ischemic stroke due to LVO treated with EVT TICI 2b, 2c, or 3 reperfusion SBP ≥ 140 mm Hg on 2 measurements within 3 h of EVT	Age ≥ 18 Acute ischemic stroke due to LVO treated with EVT TICI 2b, 2c, 3 reperfusion	Age ≥ 20 Acute ischemic stroke due to LVO treated with EVT TICI2b, 2c, or 3 reperfusion SBP ≥ 140 mm Hg on 2 measurements within a 2-min interval within 2 h of EVT
Site of Occlusion	Intracranial ICA, MCA M1, both	Any LVO (anterior or posterior circulation)	ICA, MCA M1, MCA M2	Any LVO
Exclusion Criteria	Hemorrhage during EVT Spontaneous decrease in SBP to <130 mm Hg Baseline mRS > 2	Contraindication to less or more intensive SBP management Baseline mRS > 2	Ejection fraction < 30% LVAD or ECMO Pregnancy	SBP < 140 after EVT Contraindication to antihypertensive medications sICH during or immediately after EVT Serious medical or surgical illness Pre-stroke disability (mRS 3-5)
Interventions	Intensive SBP target (100–129 mm Hg) Standard SBP target (130–185 mm Hg)	Intensive SBP target (<120 mm Hg) Standard SBP target (140–180 mm Hg)	<140 mm Hg <160 mm Hg ≤180 mm Hg	Intensive SBP target (SBP < 140 mm Hg) Conventional SBP target (SBP 140–180)
Timing for Target SBP	Achieved within 1 h, maintained for 24 h	Achieved within 1 h, maintained for 72 h	Initiated within 1 h, maintained for 24 h	Achieved within 1 h, maintained for 24 h
Primary Outcome	Rate of radiographic ICH at 24–36 h	Functional outcome at 90 d (ordinal shift in mRS)	Infarct volume at 36 (±12) hoursUtility-weighted mRS at 90 days	Functional outcome at 90 d
Results				
Mean (±SD) SBP Achieved at 1 h	Intensive group: 128 mm Hg (±11) Standard group: 138 mm Hg (±17)	Intensive group: 125 mm Hg (±18) Standard group: 143 mm Hg (±18)	Mean SBP in first 24 h: <140 mm Hg: 122 (±15) mm Hg <160 mm Hg: 130 (±18) mm Hg ≤180 mm Hg: 129 (±20) mm Hg	Intensive group: 129 mm Hg (±7.7) Conventional group:138 mm Hg (±13.6 mm)
Functional Outcomes at 90d	No difference in rate of mRS 0–1, mRS 0–2	Worse scores on mRS in intensive group OR 1.37 (95% CI 1.07–1.76)	Utility-weighted mRS: <140 mm Hg: 0.51 (95% CI 0.38–0.63) <160 mm Hg: 0.47 (95% CI 0.35–0.60) ≤180 mm Hg: 0.58 (95% CI 0.46–0.71) *p*-value for futility 0.93	Lower likelihood of functional independence (mRS 0–2) in the intensive group (39.4%) than conventional group (54.4%) Risk difference 15.1% (95% CI, −26.2% to −3.9%) aOR (0.56 [95% CI, 0.33–0.96]; *p* = 0.03)
Final infarct Volume	Not assessed	Not assessed	At 36 h: <140 mm Hg: 32.4 mL (95% CI 18 to 46.7 mL) <160 mm Hg: 50.7 mL (95% CI 33.7 to 67.7 mL) ≤180 mm Hg: 46.4 mL (95% CI 24.5 to 68.2 mL) *p*-value for futility 0.99	Intensive group: Median 18.6 mL (IQR, 6.7–62.6 mL) Conventional group 17.1 mL (IQR, 5.4–40.9 mL) *p* = 0.11
Rate of Any ICH	Intensive group: 42% Standard group: 43% OR 0.96 (95% CI 0.60–1.51; *p* = 0.84)	Intensive group: 28% Standard group: 30% OR 1.14 (95% CI 0.83–1.57; *p* = 0.43)	<140 mm Hg: 36% <160 mm Hg: 32% ≤180 mm Hg: 30%	Intensive group: 53.5% Conventional group: 52.3% (*p* = 0.93)
Definition of sICH	ECASS criteria: ICH associated with increase of ≥4 points on NIHSS at 24 h	Heidelberg criteria: ICH associated with increase in NIHSS by ≥4 points or in NIHSS subcategory by ≥2 points	ECASS criteria: ICH associated with increase of ≥4 points on NIHSS at 36 h	ECASS criteria: ICH associated with increase of ≥4 points on NIHSS at 24 h
Rate of sICH	Intensive group: 11% Standard group: 7% OR 1.68 (95% CI 0.75–3.77)	Intensive group: 6% Standard group: 6% OR 0.91 (95% CI 0.50–1.66, *p* = 0.75)	<140 mm Hg: 5% <160 mm Hg: 3% ≤180 mm Hg: 5%	Intensive group: 9% Conventional 8.1% *p* = 0.82
Early Neurologic Deterioration	No difference in change in NIHSS at 24–36 h	Shift in NIHSS toward higher scores at day 7 in intensive group OR 2.07 (95% CI 1.47–2.93)	Not reported	Not reported
Rate of Hypotension	Intensive group: 8% Standard group: 3% OR 2.53 (95% CI 0.86–7.41)	No episodes of severe hypotension in either group	No neurologic worsening related to antihypertensive treatment in any group	Intensive group: 29.7% Conventional group: 17.3% *p* = 0.02
Other Safety Outcomes	No difference in mortality	No difference in mortality or serious adverse events	In-hospital mortality 7.5% in <140 mm Hg group and <180 mm Hg groups; 15% in <160 mm Hg group	Death related to index stroke within 3 months 7.7% in intensive group vs. 5.4% in conventional group (*p* = 0.32) Malignant cerebral edema higher in intensive group (7.7%) vs. conventional group (1.3%), *p* = 0/01
Limitations	Not powered to detect difference in functional outcomes Small SBP difference achieved between groups Infarct volume and growth not assessed	High rates of intracranial atherosclerosis (intensive group 44% vs. standard group 53%) High use of IV heparin and tirofiban limit generalizability Infarct volume and growth not assessed	Small sample size Differences in baseline characteristics among groups (<140 mm Hg group had smaller infarct volume at baseline; ≤180 group had higher anticoagulant use, lower baseline NIHSS scores, and higher proportion with M2 occlusion)	Early termination The rate of time spent in the target SBP range (140–180 mm Hg) was only 42.1% in the conventional management group because of spontaneous BP decline below 140 mm Hg, which may underpower the trial The mRS primary end point was ascertained using a patient-reported rather than clinician-rated algorithm.

Abbreviations: ECASS, European Cooperative Acute Stroke Study; ECMO, extracorporeal membrane oxygenation; EVT, endovascular thrombectomy; ICA, internal carotid artery; ICH, intracerebral hemorrhage; LVAD, left ventricular assist device; LVO, large vessel occlusion; MCA M1, horizontal segment of middle cerebral artery; MCA M2, vertical portion of the middle cerebral artery in the sylvian fissure; mRS, modified Rankin score; NIHSS, National Institutes of Health Stroke Scale; RCT, randomized controlled trial; sICH, symptomatic intracerebral hemorrhage; SBP, systolic blood pressure; TICI, thrombolysis in cerebral infarction.

**Table 3:** Classification criteria for hemorrhagic transformation of ischemic stroke.

	Heidelberg Class [50]	ECASS III [14,15]	Description
Anatomic Description	1		Hemorrhagic transformation of infarct
	1a	HI1	Scattered petechial hemorrhages at infarct margin; no mass effect
	1b	HI2	Confluent petechial hemorrhages throughout infarct; no mass effect
	1c	PH1	Parenchymal hematoma involving ≤ 30% of infarcted area; mild mass effect
	2		Intracerebral hemorrhage within/beyond infarct
		PH2	Parenchymal hematoma involving > 30% of infarcted area; significant mass effect
	3		Intracerebral hemorrhage outside infarct, or extra-axial intracranial hemorrhage
	3a		Parenchymal hemorrhage outside infarct
	3b		Intraventricular hemorrhage
	3c		Subarachnoid hemorrhage
	3d		Subdural hematoma
Definition of Symptomatic ICH	Increase in NIHSS by ≥4 points Increase in NIHSS subcategory by ≥2 points Intubation, DCH, EVD placement, major medical/surgical intervention Relatedness of imaging and deterioration definite or probable as defined below: - Definite: ICH is dominant cause for deterioration - Probable: ICH is PH2, even if ischemic lesion partly responsible for deterioration - Possible: ICH is HI2, PH1, rPH, IVH, SAH, SDH, even if ischemic lesion predominantly responsible for deterioration - Unlikely: ICH is HI1	Increase in NIHSS by ≥4 points	
Relatedness of ICH to Therapeutic Intervention	Thrombolysis within 24 h Involved region/timing suggests endovascular complication Known complication from angiography Levels of certainty: - Definite: observed complication - Probable: <24 h from intervention; PH - Possible: <24 h from intervention; HI - Unrelated: no intervention within 24 h	Not defined	

Abbreviations: DHC, decompressive hemicraniectomy; EVD, external ventricular drain; ECASS, European Cooperative Acute Stroke Study; HI, hemorrhagic infarct; ICH, intracranial hemorrhage; IVH, intraventricular hemorrhage; NIHSS, National Institutes of Health Stroke Scale; PH, parenchymal hematoma; rPH, remote from infarcted brain tissue; SAH, subarachnoid hemorrhage; SDH, subdural hematoma.

**Table 4:** Summary of major studies evaluating anticoagulation after ischemic stroke.

	RAF (2015) [140]	TIMING (2022) [144]	ELAN (2023) [145]	Kimura et al. (2022) [143]
Study Design	Prospective, observational	RCT non-inferiority, open-label	RCT, open-label	Combined data from 2 prospective, observational studies (SUMARAI and RELAXED)
Setting	Europe, Asia; 29 sites	Sweden; 34 sites	15 countries; 103 sites	Japan
Dates Recruiting	Jan 2012–March 2014	April 2017–December 2020	November 2017–September 2022	September 2011–April 2016
Participants, n	1029	888	2013	1797
Inclusion Criteria	Acute ischemic stroke associated with atrial fibrillation	Acute ischemic stroke associated with atrial fibrillation	Acute ischemic stroke associated with atrial fibrillation	Acute ischemic stroke or TIA associated with atrial fibrillation - MCA stroke only for RELAXED
Exclusion Criteria	Contraindication to AC	Contraindication to DOAC	Parenchymal hematoma Anticoagulated at baseline	Prosthetic valve Clinically significant hemorrhage
Timing of AC	At discretion of treating physician	Early: ≤4 days Delayed: 5–10 days	Early: - Minor/Moderate stroke: ^Ω^ < 48 h - Major stroke: Day 6–7 Delayed: - Minor stroke: Day 3–4 - Moderate: Day 6–7 - Major stroke: Day 12–14	Early: patients starting DOAC earlier than median initiation day in each subgroup - TIA: <2 day - Mild (NIHSS 0–7): <3 days - Moderate (NIHSS 8–15): <4 days - Severe (NIHSS ≥ 16): <5 days
Type of AC	Any; at discretion of treating physician	Apixaban, Dabigatran, Edoxoban, Rivaroxaban Choice of DOAC at discretion of treating physician	Any locally approved DOAC	RELAXED: Rivaroxaban SAMURAI: Dabigatran, Rivaroxaban, Apixaban
Median NIHSS (IQR)	Mean NIHSS 9.2 ± 7.3	Early: 4 (2–9) Delayed: 4 (2–8)	Early: 5 (2–12) Delayed: 5 (2–11)	Early: 8 (2–16) Delayed: 6 (2–15)
Thrombolysis	Not reported; 22.3% with thrombolysis or thrombectomy	Early: 29.3% Delayed: 27.4%	Early: 39.7% Delayed: 38.2%	Early: 29% Delayed: 22%
Thrombectomy	Not reported; 22.3% with thrombolysis or thrombectomy	Early: 14.4% Delayed: 12.8%	Early: 21.0% Delayed: 23.5%	Early: 17% Delayed: 10%
Primary Outcome	Composite of ischemic stroke/TIA, symptomatic systemic embolism, sICH, major extracranial bleeding	Composite of ischemic stroke, sICH, all-cause mortality	Composite of ischemic stroke, systemic embolism, major extracranial bleeding, sICH, vascular death	Composite of ischemic stroke, systemic embolism
Timing of Outcome Assessment	90 days	90 days	30 days and 90 days	90 days
Results at 90 Days				
Rate of Primary Outcome	12.6% overall AC: 11.7% No AC: 14.4%	Early: 6.9% Delayed: 8.7% Risk difference −1.8% (95% CI −5.3% to 1.7%, *p* = 0.004 for non-inferiority)	Early: 3.7% Delayed: 5.6% Risk difference −1.9% (95% CI −3.8% to −0.02%)	Early: 1.9% Delayed: 3.9% HR 0.50 (95% CI 0.27–0.89)
Rate of Ischemic Stroke	7.6% with stroke, TIA, or systemic embolism	Early: 3.1% Delayed: 4.6%	Early:1.4% Delayed: 2.5%	Early: 1.7% Delayed: 3.2%
Rate of sICH	3.6%	No sICH in either group	Early: 0.2% Delayed: 0.2%	Rate for any ICH Early: 0.3% Delayed: 0.4%
Rate of Systemic Embolism	Not reported	Not reported	Early: 0.4% Delayed: 1.0%	Early: 0.3% Delayed: 0.6%
Rate of Major Bleeding	1.4%	Early: 1.6% Delayed: 0.7% (Includes asymptomatic ICH)	Early: 0.3% Delayed: 0.8% (Excludes intracranial bleeding)	Early: 0.8% Delayed: 1.0%
Rate of All-Cause Mortality	10.9%	Early: 4.7% Delayed: 5.7%	Early: 1.8% Delayed: 1.7% (Vascular death only)	Early: 2.2% Delayed: 2.2%
Optimal Timing for AC	4–14 days (vs. < 4 or > 14 days) HR 0.53 (95% CI 0.30–0.93)	Early initiation non-inferior to delayed start of DOAC	No conclusion drawn regarding superiority or non-inferiority or early vs. late initiation of AC	Early initiation associated with better efficacy and similar safety
Limitations	Observational study No brain imaging data on lesion size	Low recruitment No brain imaging data on lesion size	Patients anticoagulated at baseline excluded Low median NIHSS	Observational study Higher rates of thrombolysis and EVT in early group Dose of rivaroxaban in Japan is 10–15 mg, lower than many other countries NIHSS rather than infarct size used to stratify groups

^Ω^ Definition of infarct size: minor stroke defined as infarct ≤ 1.5 cm; moderate stroke as infarct in the distribution of a cortical branch of MCA, ACA, or MCA; major stroke as brainstem or cerebellar infarcts > 1.5 cm, or infarct in distribution of 2+ branches of MCA or entire territory of ACA or PCA. Abbreviations: AC, anticoagulation; DOAC, direct oral anticoagulant; ELAN, Early versus Later Anticoagulation for Stroke with Atrial Fibrillation; EVT, endovascular thrombectomy; ICH, intracerebral hemorrhage; MCA, middle cerebral artery; NIHSS, National Institutes of Health Stroke Scale; sICH symptomatic intracerebral hemorrhage; TIA, transient ischemic attack; TIMING, Timing of Oral Anticoagulant Therapy in Acute Ischemic Stroke with Atrial Fibrillation.

## References

[1] Sarraj A., Hassan A.E., Savitz S., Sitton C., Grotta J., Chen P., Cai C., Cutter G., Imam B., Reddy S. (2019). Outcomes of Endovascular Thrombectomy vs. Medical Management Alone in Patients with Large Ischemic Cores: A Secondary Analysis of the Optimizing Patient’s Selection for Endovascular Treatment in Acute Ischemic Stroke (SELECT) Study. JAMA Neurol..

[2] Reinink H., Juttler E., Hacke W., Hofmeijer J., Vicaut E., Vahedi K., Slezins J., Su Y., Fan L., Kumral E. (2021). Surgical Decompression for Space-Occupying Hemispheric Infarction: A Systematic Review and Individual Patient Meta-analysis of Randomized Clinical Trials. JAMA Neurol..

[3] Zaidi S.F., Aghaebrahim A., Urra X., Jumaa M.A., Jankowitz B., Hammer M., Nogueira R., Horowitz M., Reddy V., Jovin T.G. (2012). Final infarct volume is a stronger predictor of outcome than recanalization in patients with proximal middle cerebral artery occlusion treated with endovascular therapy. Stroke.

[4] Sims J.R., Gharai L.R., Schaefer P.W., Vangel M., Rosenthal E.S., Lev M.H., Schwamm L.H. (2009). ABC/2 for rapid clinical estimate of infarct, perfusion, and mismatch volumes. Neurology.

[5] Lansberg M.G., Lee J., Christensen S., Straka M., De Silva D.A., Mlynash M., Campbell B.C., Bammer R., Olivot J.M., Desmond P. (2011). RAPID automated patient selection for reperfusion therapy: A pooled analysis of the Echoplanar Imaging Thrombolytic Evaluation Trial (EPITHET) and the Diffusion and Perfusion Imaging Evaluation for Understanding Stroke Evolution (DEFUSE) Study. Stroke.

[6] Sarraj A., Campbell B., Ribo M., Hussain M.S., Chen M., Abraham M.G., Lansberg M.G., Mendes Pereira V., Blackburn S., Sitton C.W. (2021). SELECTion criteria for large core trials: Dogma or data?. J. Neurointerv. Surg..

[7] Jadhav A.P., Hacke W., Dippel D.W.J., Simonsen C.Z., Costalat V., Fiehler J., Thomalla G., Bendszus M., Andersson T., Mattle H.P. (2021). Select wisely: The ethical challenge of defining large core with perfusion in the early time window. J. Neurointerv. Surg..

[8] Yoo A.J., Verduzco L.A., Schaefer P.W., Hirsch J.A., Rabinov J.D., Gonzalez R.G. (2009). MRI-based selection for intra-arterial stroke therapy: Value of pretreatment diffusion-weighted imaging lesion volume in selecting patients with acute stroke who will benefit from early recanalization. Stroke.

[9] Parsons M.W., Christensen S., McElduff P., Levi C.R., Butcher K.S., De Silva D.A., Ebinger M., Barber P.A., Bladin C., Donnan G.A. (2010). Pretreatment diffusion- and perfusion-MR lesion volumes have a crucial influence on clinical response to stroke thrombolysis. J. Cereb. Blood Flow Metab..

[10] Thomalla G.J., Kucinski T., Schoder V., Fiehler J., Knab R., Zeumer H., Weiller C., Rother J. (2003). Prediction of malignant middle cerebral artery infarction by early perfusion- and diffusion-weighted magnetic resonance imaging. Stroke.

[11] Wu S., Yuan R., Wang Y., Wei C., Zhang S., Yang X., Wu B., Liu M. (2018). Early Prediction of Malignant Brain Edema after Ischemic Stroke. Stroke.

[12] Geuskens R.R., Borst J., Lucas M., Boers A.M., Berkhemer O.A., Roos Y.B., van Walderveen M.A., Jenniskens S.F., van Zwam W.H., Dippel D.W. (2015). Characteristics of Misclassified CT Perfusion Ischemic Core in Patients with Acute Ischemic Stroke. PLoS ONE.

[13] National Institute of Neurological Disorders, Stroke rt-PA Stroke Study Group (1995). Tissue plasminogen activator for acute ischemic stroke. N. Engl. J. Med..

[14] Hacke W., Kaste M., Fieschi C., Toni D., Lesaffre E., von Kummer R., Boysen G., Bluhmki E., Hoxter G., Mahagne M.H. (1995). Intravenous thrombolysis with recombinant tissue plasminogen activator for acute hemispheric stroke. The European Cooperative Acute Stroke Study (ECASS). JAMA.

[15] Hacke W., Kaste M., Bluhmki E., Brozman M., Davalos A., Guidetti D., Larrue V., Lees K.R., Medeghri Z., Machnig T. (2008). Thrombolysis with alteplase 3 to 4.5 hours after acute ischemic stroke. N. Engl. J. Med..

[16] Anadani M., Almallouhi E., Maier I., Al Kasab S., Jabbour P., Kim J.T., Wolfe S.Q., Rai A., Starke R.M., Psychogios M.N. (2023). Effect of intravenous thrombolysis before endovascular therapy on outcomes in patients with large core infarct. J. Neurointerv. Surg..

[17] Chen C., Parsons M.W., Levi C.R., Spratt N.J., Miteff F., Lin L., Cheng X., Lou M., Kleinig T., Butcher K. (2019). Exploring the relationship between ischemic core volume and clinical outcomes after thrombectomy or thrombolysis. Neurology.

[18] Leslie-Mazwi T.M., Altschul D., Simonsen C.Z. (2021). Thrombectomy for Patients with Large Infarct Core in Practice: Where Should the Pendulum Swing?. Stroke.

[19] Berkhemer O.A., Fransen P.S., Beumer D., van den Berg L.A., Lingsma H.F., Yoo A.J., Schonewille W.J., Vos J.A., Nederkoorn P.J., Wermer M.J. (2015). A randomized trial of intraarterial treatment for acute ischemic stroke. N. Engl. J. Med..

[20] Goyal M., Demchuk A.M., Menon B.K., Eesa M., Rempel J.L., Thornton J., Roy D., Jovin T.G., Willinsky R.A., Sapkota B.L. (2015). Randomized assessment of rapid endovascular treatment of ischemic stroke. N. Engl. J. Med..

[21] Jovin T.G., Chamorro A., Cobo E., de Miquel M.A., Molina C.A., Rovira A., San Roman L., Serena J., Abilleira S., Ribo M. (2015). Thrombectomy within 8 hours after symptom onset in ischemic stroke. N. Engl. J. Med..

[22] Saver J.L., Goyal M., Bonafe A., Diener H.C., Levy E.I., Pereira V.M., Albers G.W., Cognard C., Cohen D.J., Hacke W. (2015). Stent-retriever thrombectomy after intravenous t-PA vs. t-PA alone in stroke. N. Engl. J. Med..

[23] Campbell B.C., Mitchell P.J., Kleinig T.J., Dewey H.M., Churilov L., Yassi N., Yan B., Dowling R.J., Parsons M.W., Oxley T.J. (2015). Endovascular therapy for ischemic stroke with perfusion-imaging selection. N. Engl. J. Med..

[24] Nogueira R.G., Jadhav A.P., Haussen D.C., Bonafe A., Budzik R.F., Bhuva P., Yavagal D.R., Ribo M., Cognard C., Hanel R.A. (2018). Thrombectomy 6 to 24 Hours after Stroke with a Mismatch between Deficit and Infarct. N. Engl. J. Med..

[25] Albers G.W., Marks M.P., Kemp S., Christensen S., Tsai J.P., Ortega-Gutierrez S., McTaggart R.A., Torbey M.T., Kim-Tenser M., Leslie-Mazwi T. (2018). Thrombectomy for Stroke at 6 to 16 Hours with Selection by Perfusion Imaging. N. Engl. J. Med..

[26] Yoshimura S., Sakai N., Yamagami H., Uchida K., Beppu M., Toyoda K., Matsumaru Y., Matsumoto Y., Kimura K., Takeuchi M. (2022). Endovascular Therapy for Acute Stroke with a Large Ischemic Region. N. Engl. J. Med..

[27] Fayad P. (2023). Improved Prospects for Thrombectomy in Large Ischemic Stroke. N. Engl. J. Med..

[28] Sarraj A., Hassan A.E., Abraham M.G., Ortega-Gutierrez S., Kasner S.E., Hussain M.S., Chen M., Blackburn S., Sitton C.W., Churilov L. (2023). Trial of Endovascular Thrombectomy for Large Ischemic Strokes. N. Engl. J. Med..

[29] Huo X., Ma G., Tong X., Zhang X., Pan Y., Nguyen T.N., Yuan G., Han H., Chen W., Wei M. (2023). Trial of Endovascular Therapy for Acute Ischemic Stroke with Large Infarct. N. Engl. J. Med..

[30] Goyal M., Menon B.K., van Zwam W.H., Dippel D.W., Mitchell P.J., Demchuk A.M., Davalos A., Majoie C.B., van der Lugt A., de Miquel M.A. (2016). Endovascular thrombectomy after large-vessel ischaemic stroke: A meta-analysis of individual patient data from five randomised trials. Lancet.

[31] Paulson O.B., Strandgaard S., Edvinsson L. (1990). Cerebral autoregulation. Cerebrovasc. Brain Metab. Rev..

[32] Leonardi-Bee J., Bath P.M., Phillips S.J., Sandercock P.A.G., IST Collaborative Group (2002). Blood pressure and clinical outcomes in the International Stroke Trial. Stroke.

[33] Okumura K., Ohya Y., Maehara A., Wakugami K., Iseki K., Takishita S. (2005). Effects of blood pressure levels on case fatality after acute stroke. J. Hypertens..

[34] Martins A.I., Sargento-Freitas J., Silva F., Jesus-Ribeiro J., Correia I., Gomes J.P., Aguiar-Goncalves M., Cardoso L., Machado C., Rodrigues B. (2016). Recanalization Modulates Association Between Blood Pressure and Functional Outcome in Acute Ischemic Stroke. Stroke.

[35] John S., Hazaa W., Uchino K., Hussain M.S. (2017). Timeline of blood pressure changes after intra-arterial therapy for acute ischemic stroke based on recanalization status. J. Neurointerv. Surg..

[36] Matusevicius M., Cooray C., Bottai M., Mazya M., Tsivgoulis G., Nunes A.P., Moreira T., Ollikainen J., Tassi R., Strbian D. (2020). Blood Pressure after Endovascular Thrombectomy: Modeling for Outcomes Based on Recanalization Status. Stroke.

[37] Morris N.A., Jindal G., Chaturvedi S. (2023). Intensive Blood Pressure Control after Mechanical Thrombectomy for Acute Ischemic Stroke. Stroke.

[38] Samuels N., van de Graaf R.A., van den Berg C.A.L., Uniken Venema S.M., Bala K., van Doormaal P.J., van der Steen W., Witvoet E., Boiten J., den Hertog H. (2021). Blood Pressure in the First 6 Hours Following Endovascular Treatment for Ischemic Stroke Is Associated with Outcome. Stroke.

[39] Mazighi M., Richard S., Lapergue B., Sibon I., Gory B., Berge J., Consoli A., Labreuche J., Olivot J.M., Broderick J. (2021). Safety and efficacy of intensive blood pressure lowering after successful endovascular therapy in acute ischaemic stroke (BP-TARGET): A multicentre, open-label, randomised controlled trial. Lancet Neurol..

[40] Yang P., Song L., Zhang Y., Zhang X., Chen X., Li Y., Sun L., Wan Y., Billot L., Li Q. (2022). Intensive blood pressure control after endovascular thrombectomy for acute ischaemic stroke (ENCHANTED2/MT): A multicentre, open-label, blinded-endpoint, randomised controlled trial. Lancet.

[41] Mistry E.A., Hart K.W., Davis L.T., Gao Y., Prestigiacomo C.J., Mittal S., Mehta T., LaFever H., Harker P., Wilson-Perez H.E. (2023). Blood Pressure Management after Endovascular Therapy for Acute Ischemic Stroke: The BEST-II Randomized Clinical Trial. JAMA.

[42] Nam H.S., Kim Y.D., Heo J., Lee H., Jung J.W., Choi J.K., Lee I.H., Lim I.H., Hong S.H., Baik M. (2023). Intensive vs. Conventional Blood Pressure Lowering after Endovascular Thrombectomy in Acute Ischemic Stroke: The OPTIMAL-BP Randomized Clinical Trial. JAMA.

[43] Powers W.J., Rabinstein A.A., Ackerson T., Adeoye O.M., Bambakidis N.C., Becker K., Biller J., Brown M., Demaerschalk B.M., Hoh B. (2019). Guidelines for the Early Management of Patients with Acute Ischemic Stroke: 2019 Update to the 2018 Guidelines for the Early Management of Acute Ischemic Stroke: A Guideline for Healthcare Professionals From the American Heart Association/American Stroke Association. Stroke.

[44] Mistry E.A., Mehta T., Mistry A., Arora N., Starosciak A.K., De Los Rios La Rosa F., Siegler J.E., Chitale R., Anadani M., Yaghi S. (2020). Blood Pressure Variability and Neurologic Outcome after Endovascular Thrombectomy: A Secondary Analysis of the BEST Study. Stroke.

[45] Webb A.J., Rothwell P.M. (2011). Effect of dose and combination of antihypertensives on interindividual blood pressure variability: A systematic review. Stroke.

[46] Regenhardt R.W., Das A.S., Stapleton C.J., Chandra R.V., Rabinov J.D., Patel A.B., Hirsch J.A., Leslie-Mazwi T.M. (2017). Blood Pressure and Penumbral Sustenance in Stroke from Large Vessel Occlusion. Front. Neurol..

[47] Larrue V., von Kummer R., del Zoppo G., Bluhmki E. (1997). Hemorrhagic transformation in acute ischemic stroke. Potential contributing factors in the European Cooperative Acute Stroke Study. Stroke.

[48] Larrue V., von Kummer R.R., Muller A., Bluhmki E. (2001). Risk factors for severe hemorrhagic transformation in ischemic stroke patients treated with recombinant tissue plasminogen activator: A secondary analysis of the European-Australasian Acute Stroke Study (ECASS II). Stroke.

[49] Fiorelli M., Bastianello S., von Kummer R., del Zoppo G.J., Larrue V., Lesaffre E., Ringleb A.P., Lorenzano S., Manelfe C., Bozzao L. (1999). Hemorrhagic transformation within 36 hours of a cerebral infarct: Relationships with early clinical deterioration and 3-month outcome in the European Cooperative Acute Stroke Study I (ECASS I) cohort. Stroke.

[50] von Kummer R., Broderick J.P., Campbell B.C., Demchuk A., Goyal M., Hill M.D., Treurniet K.M., Majoie C.B., Marquering H.A., Mazya M.V. (2015). The Heidelberg Bleeding Classification: Classification of Bleeding Events after Ischemic Stroke and Reperfusion Therapy. Stroke.

[51] Whiteley W.N., Emberson J., Lees K.R., Blackwell L., Albers G., Bluhmki E., Brott T., Cohen G., Davis S., Donnan G. (2016). Risk of intracerebral haemorrhage with alteplase after acute ischaemic stroke: A secondary analysis of an individual patient data meta-analysis. Lancet Neurol..

[52] Whiteley W.N., Slot K.B., Fernandes P., Sandercock P., Wardlaw J. (2012). Risk factors for intracranial hemorrhage in acute ischemic stroke patients treated with recombinant tissue plasminogen activator: A systematic review and meta-analysis of 55 studies. Stroke.

[53] Yaghi S., Willey J.Z., Cucchiara B., Goldstein J.N., Gonzales N.R., Khatri P., Kim L.J., Mayer S.A., Sheth K.N., Schwamm L.H. (2017). Treatment and Outcome of Hemorrhagic Transformation after Intravenous Alteplase in Acute Ischemic Stroke: A Scientific Statement for Healthcare Professionals From the American Heart Association/American Stroke Association. Stroke.

[54] Frontera J.A., Lewin J.J., Rabinstein A.A., Aisiku I.P., Alexandrov A.W., Cook A.M., del Zoppo G.J., Kumar M.A., Peerschke E.I., Stiefel M.F. (2016). Guideline for Reversal of Antithrombotics in Intracranial Hemorrhage: A Statement for Healthcare Professionals from the Neurocritical Care Society and Society of Critical Care Medicine. Neurocrit. Care.

[55] Yaghi S., Boehme A.K., Dibu J., Leon Guerrero C.R., Ali S., Martin-Schild S., Sands K.A., Noorian A.R., Blum C.A., Chaudhary S. (2015). Treatment and Outcome of Thrombolysis-Related Hemorrhage: A Multicenter Retrospective Study. JAMA Neurol..

[56] Hacke W., Schwab S., Horn M., Spranger M., De Georgia M., von Kummer R. (1996). ‘Malignant’ middle cerebral artery territory infarction: Clinical course and prognostic signs. Arch. Neurol..

[57] Hofmeijer J., Algra A., Kappelle L.J., van der Worp H.B. (2008). Predictors of life-threatening brain edema in middle cerebral artery infarction. Cerebrovasc. Dis..

[58] Wijdicks E.F., Sheth K.N., Carter B.S., Greer D.M., Kasner S.E., Kimberly W.T., Schwab S., Smith E.E., Tamargo R.J., Wintermark M. (2014). Recommendations for the management of cerebral and cerebellar infarction with swelling: A statement for healthcare professionals from the American Heart Association/American Stroke Association. Stroke.

[59] Wu S., Wang Y., Yuan R., Guo F., Yang D., Li Z., Wu B., Wang C., Duan J., Ling T. (2023). Predicting the emergence of malignant brain oedema in acute ischaemic stroke: A prospective multicentre study with development and validation of predictive modelling. EClinicalMedicine.

[60] Barber P.A., Demchuk A.M., Zhang J., Kasner S.E., Hill M.D., Berrouschot J., Schmutzhard E., Harms L., Verro P., Krieger D. (2003). Computed tomographic parameters predicting fatal outcome in large middle cerebral artery infarction. Cerebrovasc. Dis..

[61] Park J., Goh D.H., Sung J.K., Hwang Y.H., Kang D.H., Kim Y. (2012). Timely assessment of infarct volume and brain atrophy in acute hemispheric infarction for early surgical decompression: Strict cutoff criteria with high specificity. Acta Neurochir..

[62] Poca M.A., Benejam B., Sahuquillo J., Riveiro M., Frascheri L., Merino M.A., Delgado P., Alvarez-Sabin J. (2010). Monitoring intracranial pressure in patients with malignant middle cerebral artery infarction: Is it useful?. J. Neurosurg..

[63] Schwab S., Aschoff A., Spranger M., Albert F., Hacke W. (1996). The value of intracranial pressure monitoring in acute hemispheric stroke. Neurology.

[64] Cook A.M., Morgan Jones G., Hawryluk G.W.J., Mailloux P., McLaughlin D., Papangelou A., Samuel S., Tokumaru S., Venkatasubramanian C., Zacko C. (2020). Guidelines for the Acute Treatment of Cerebral Edema in Neurocritical Care Patients. Neurocrit. Care.

[65] Frank J.I., Schumm L.P., Wroblewski K., Chyatte D., Rosengart A.J., Kordeck C., Thisted R.A., He A.T. (2014). Hemicraniectomy and durotomy upon deterioration from infarction-related swelling trial: Randomized pilot clinical trial. Stroke.

[66] Berrouschot J., Sterker M., Bettin S., Koster J., Schneider D. (1998). Mortality of space-occupying (‘malignant’) middle cerebral artery infarction under conservative intensive care. Intensive Care Med..

[67] Manno E.M., Adams R.E., Derdeyn C.P., Powers W.J., Diringer M.N. (1999). The effects of mannitol on cerebral edema after large hemispheric cerebral infarct. Neurology.

[68] Chen C.H., Toung T.J., Sapirstein A., Bhardwaj A. (2006). Effect of duration of osmotherapy on blood-brain barrier disruption and regional cerebral edema after experimental stroke. J. Cereb. Blood Flow Metab..

[69] Candelise L., Colombo A., Spinnler H. (1975). Therapy against brain swelling in stroke patients. A retrospective clinical study on 227 patients. Stroke.

[70] Bereczki D., Mihalka L., Szatmari S., Fekete K., Di Cesar D., Fulesdi B., Csiba L., Fekete I. (2003). Mannitol use in acute stroke: Case fatality at 30 days and 1 year. Stroke.

[71] Koenig M.A., Bryan M., Lewin J.L., Mirski M.A., Geocadin R.G., Stevens R.D. (2008). Reversal of transtentorial herniation with hypertonic saline. Neurology.

[72] Mohney N., Alkhatib O., Koch S., O’Phelan K., Merenda A. (2020). What is the Role of Hyperosmolar Therapy in Hemispheric Stroke Patients?. Neurocrit. Care.

[73] Vahedi K., Vicaut E., Mateo J., Kurtz A., Orabi M., Guichard J.P., Boutron C., Couvreur G., Rouanet F., Touze E. (2007). Sequential-design, multicenter, randomized, controlled trial of early decompressive craniectomy in malignant middle cerebral artery infarction (DECIMAL Trial). Stroke.

[74] Juttler E., Schwab S., Schmiedek P., Unterberg A., Hennerici M., Woitzik J., Witte S., Jenetzky E., Hacke W., Group D.S. (2007). Decompressive Surgery for the Treatment of Malignant Infarction of the Middle Cerebral Artery (DESTINY): A randomized, controlled trial. Stroke.

[75] Hofmeijer J., Kappelle L.J., Algra A., Amelink G.J., van Gijn J., van der Worp H.B., HAMLET investigators (2009). Surgical decompression for space-occupying cerebral infarction (the Hemicraniectomy after Middle Cerebral Artery infarction with Life-threatening Edema Trial [HAMLET]): A multicentre, open, randomised trial. Lancet Neurol..

[76] Slezins J., Keris V., Bricis R., Millers A., Valeinis E., Stukens J., Minibajeva O. (2012). Preliminary results of randomized controlled study on decompressive craniectomy in treatment of malignant middle cerebral artery stroke. Medicina.

[77] Zhao J., Su Y.Y., Zhang Y., Zhang Y.Z., Zhao R., Wang L., Gao R., Chen W., Gao D. (2012). Decompressive hemicraniectomy in malignant middle cerebral artery infarct: A randomized controlled trial enrolling patients up to 80 years old. Neurocrit. Care.

[78] Juttler E., Bosel J., Amiri H., Schiller P., Limprecht R., Hacke W., Unterberg A., Group D.I.S. (2011). DESTINY II: DEcompressive Surgery for the Treatment of malignant INfarction of the middle cerebral arterY II. Int. J. Stroke.

[79] Chua A., Buckley B., Capitan M., Jamora R. (2015). Hemicraniectomy for malignant middle cerebral artery infarction (HeMMI): A Hemicraniectomy for malignant middle cerebral artery infarction (HeMMI): Randomised controlled clinical trail of decompressive hemicraniectomy with standardised medical care versus standardised medical care alone. Acta Med. Philipp..

[80] Vahedi K., Hofmeijer J., Juettler E., Vicaut E., George B., Algra A., Amelink G.J., Schmiedeck P., Schwab S., Rothwell P.M. (2007). Early decompressive surgery in malignant infarction of the middle cerebral artery: A pooled analysis of three randomised controlled trials. Lancet Neurol..

[81] Juttler E., Unterberg A., Woitzik J., Bosel J., Amiri H., Sakowitz O.W., Gondan M., Schiller P., Limprecht R., Luntz S. (2014). Hemicraniectomy in older patients with extensive middle-cerebral-artery stroke. N. Engl. J. Med..

[82] Dasenbrock H.H., Robertson F.C., Vaitkevicius H., Aziz-Sultan M.A., Guttieres D., Dunn I.F., Du R., Gormley W.B. (2017). Timing of Decompressive Hemicraniectomy for Stroke: A Nationwide Inpatient Sample Analysis. Stroke.

[83] Gupta R., Connolly E.S., Mayer S., Elkind M.S. (2004). Hemicraniectomy for massive middle cerebral artery territory infarction: A systematic review. Stroke.

[84] Schwab S., Steiner T., Aschoff A., Schwarz S., Steiner H.H., Jansen O., Hacke W. (1998). Early hemicraniectomy in patients with complete middle cerebral artery infarction. Stroke.

[85] Cho D.Y., Chen T.C., Lee H.C. (2003). Ultra-early decompressive craniectomy for malignant middle cerebral artery infarction. Surg. Neurol..

[86] Smania N., Gandolfi M., Aglioti S.M., Girardi P., Fiaschi A., Girardi F. (2010). How long is the recovery of global aphasia? Twenty-five years of follow-up in a patient with left hemisphere stroke. Neurorehabil. Neural Repair..

[87] Tanrikulu L., Oez-Tanrikulu A., Weiss C., Scholz T., Schiefer J., Clusmann H., Schubert G.A. (2015). The bigger, the better? About the size of decompressive hemicraniectomies. Clin. Neurol. Neurosurg..

[88] Koo J., Lee J., Lee S.H., Moon J.H., Yang S.Y., Cho K.T. (2021). Does the Size of Unilateral Decompressive Craniectomy Impact Clinical Outcomes in Patients with Intracranial Mass Effect after Severe Traumatic Brain Injury?. Korean J. Neurotrauma.

[89] Lin J., Frontera J.A. (2021). Decompressive Hemicraniectomy for Large Hemispheric Strokes. Stroke.

[90] Yerram S., Katyal N., Premkumar K., Nattanmai P., Newey C.R. (2018). Seizure prophylaxis in the neuroscience intensive care unit. J. Intensive Care.

[91] Greenhalgh J., Weston J., Dundar Y., Nevitt S.J., Marson A.G. (2018). Antiepileptic drugs as prophylaxis for postcraniotomy seizures. Cochrane Database Syst. Rev..

[92] Pulman J., Greenhalgh J., Marson A.G. (2013). Antiepileptic drugs as prophylaxis for post-craniotomy seizures. Cochrane Database Syst. Rev..

[93] De Cola M.C., Corallo F., Pria D., Lo Buono V., Calabro R.S. (2018). Timing for cranioplasty to improve neurological outcome: A systematic review. Brain Behav..

[94] O’Collins V.E., Macleod M.R., Donnan G.A., Horky L.L., van der Worp B.H., Howells D.W. (2006). 1026 experimental treatments in acute stroke. Ann. Neurol..

[95] Sandercock P.A., Soane T. (2011). Corticosteroids for acute ischaemic stroke. Cochrane Database Syst. Rev..

[96] Bardutzky J., Schwab S. (2007). Antiedema therapy in ischemic stroke. Stroke.

[97] Els T., Oehm E., Voigt S., Klisch J., Hetzel A., Kassubek J. (2006). Safety and therapeutical benefit of hemicraniectomy combined with mild hypothermia in comparison with hemicraniectomy alone in patients with malignant ischemic stroke. Cerebrovasc. Dis..

[98] Jeong H.Y., Chang J.Y., Yum K.S., Hong J.H., Jeong J.H., Yeo M.J., Bae H.J., Han M.K., Lee K. (2016). Extended Use of Hypothermia in Elderly Patients with Malignant Cerebral Edema as an Alternative to Hemicraniectomy. J. Stroke.

[99] Schwab S., Schwarz S., Spranger M., Keller E., Bertram M., Hacke W. (1998). Moderate hypothermia in the treatment of patients with severe middle cerebral artery infarction. Stroke.

[100] Park H.S., Choi J.H. (2018). Safety and Efficacy of Hypothermia (34 degrees C) after Hemicraniectomy for Malignant MCA Infarction. J. Korean Neurosurg. Soc..

[101] Neugebauer H., Schneider H., Bosel J., Hobohm C., Poli S., Kollmar R., Sobesky J., Wolf S., Bauer M., Tittel S. (2019). Outcomes of Hypothermia in Addition to Decompressive Hemicraniectomy in Treatment of Malignant Middle Cerebral Artery Stroke: A Randomized Clinical Trial. JAMA Neurol..

[102] A Trial of Intravascular Hypothermia Therapy in Acute Ischemic Stroke Patients. https://classic.clinicaltrials.gov/ct2/show/NCT04695236.

[103] REperfusion with Cooling in CerebraL Acute IscheMia II (RECCLAIM-II). https://www.clinicaltrials.gov/study/NCT03804060?cond=Ischemic%20Stroke&intr=hypothermia&page=2&rank=13.

[104] Castillo J., Davalos A., Marrugat J., Noya M. (1998). Timing for fever-related brain damage in acute ischemic stroke. Stroke.

[105] Saini M., Saqqur M., Kamruzzaman A., Lees K.R., Shuaib A., Investigators V. (2009). Effect of hyperthermia on prognosis after acute ischemic stroke. Stroke.

[106] Els T., Klisch J., Orszagh M., Hetzel A., Schulte-Monting J., Schumacher M., Lucking C.H. (2002). Hyperglycemia in patients with focal cerebral ischemia after intravenous thrombolysis: Influence on clinical outcome and infarct size. Cerebrovasc. Dis..

[107] Mi D., Wang P., Yang B., Pu Y., Yang Z., Liu L. (2018). Correlation of hyperglycemia with mortality after acute ischemic stroke. Ther. Adv. Neurol. Disord..

[108] Bruno A., Levine S.R., Frankel M.R., Brott T.G., Lin Y., Tilley B.C., Lyden P.D., Broderick J.P., Kwiatkowski T.G., Fineberg S.E. (2002). Admission glucose level and clinical outcomes in the NINDS rt-PA Stroke Trial. Neurology.

[109] Simard J.M., Chen M., Tarasov K.V., Bhatta S., Ivanova S., Melnitchenko L., Tsymbalyuk N., West G.A., Gerzanich V. (2006). Newly expressed SUR1-regulated NC(Ca-ATP) channel mediates cerebral edema after ischemic stroke. Nat. Med..

[110] Simard J.M., Tsymbalyuk N., Tsymbalyuk O., Ivanova S., Yurovsky V., Gerzanich V. (2010). Glibenclamide is superior to decompressive craniectomy in a rat model of malignant stroke. Stroke.

[111] Simard J.M., Yurovsky V., Tsymbalyuk N., Melnichenko L., Ivanova S., Gerzanich V. (2009). Protective effect of delayed treatment with low-dose glibenclamide in three models of ischemic stroke. Stroke.

[112] Kimberly W.T., Bevers M.B., von Kummer R., Demchuk A.M., Romero J.M., Elm J.J., Hinson H.E., Molyneaux B.J., Simard J.M., Sheth K.N. (2018). Effect of IV glyburide on adjudicated edema endpoints in the GAMES-RP Trial. Neurology.

[113] Sheth K.N., Elm J.J., Molyneaux B.J., Hinson H., Beslow L.A., Sze G.K., Ostwaldt A.C., Del Zoppo G.J., Simard J.M., Jacobson S. (2016). Safety and efficacy of intravenous glyburide on brain swelling after large hemispheric infarction (GAMES-RP): A randomised, double-blind, placebo-controlled phase 2 trial. Lancet Neurol..

[114] Sheth K.N., Simard J.M., Elm J., Kronenberg G., Kunte H., Kimberly W.T. (2016). Human Data Supporting Glyburide in Ischemic Stroke. Acta Neurochir. Suppl..

[115] Sheth K.N., Petersen N.H., Cheung K., Elm J.J., Hinson H.E., Molyneaux B.J., Beslow L.A., Sze G.K., Simard J.M., Kimberly W.T. (2018). Long-Term Outcomes in Patients Aged </=70 Years with Intravenous Glyburide from the Phase II GAMES-RP Study of Large Hemispheric Infarction: An Exploratory Analysis. Stroke.

[116] Phase 3 Study to Evaluate the Efficacy and Safety of Intravenous BIIB093 (Glibenclamide) for Severe Cerebral Edema Following Large Hemispheric Infarction (CHARM). https://classic.clinicaltrials.gov/ct2/show/NCT02864953.

[117] Yao Y., Zhang Y., Liao X., Yang R., Lei Y., Luo J. (2020). Potential Therapies for Cerebral Edema after Ischemic Stroke: A Mini Review. Front. Aging Neurosci..

[118] Sen A., Jette N., Husain M., Sander J.W. (2020). Epilepsy in older people. Lancet.

[119] Hsu C.J., Weng W.C., Peng S.S., Lee W.T. (2014). Early-onset seizures are correlated with late-onset seizures in children with arterial ischemic stroke. Stroke.

[120] Graham N.S., Crichton S., Koutroumanidis M., Wolfe C.D., Rudd A.G. (2013). Incidence and associations of poststroke epilepsy: The prospective South London Stroke Register. Stroke.

[121] Tanaka T., Ihara M. (2017). Post-stroke epilepsy. Neurochem. Int..

[122] Fisher R.S., Acevedo C., Arzimanoglou A., Bogacz A., Cross J.H., Elger C.E., Engel J., Forsgren L., French J.A., Glynn M. (2014). ILAE official report: A practical clinical definition of epilepsy. Epilepsia.

[123] Ferreira-Atuesta C., Dohler N., Erdelyi-Canavese B., Felbecker A., Siebel P., Scherrer N., Bicciato G., Schweizer J., Sinka L., Imbach L.L. (2021). Seizures after Ischemic Stroke: A Matched Multicenter Study. Ann. Neurol..

[124] Belcastro V., Brigo F., Ferlazzo E., Gasparini S., Mastroianni G., Cianci V., Lattanzi S., Silvestrini M., Versino M., Banfi P. (2020). Incidence of early poststroke seizures during reperfusion therapies in patients with acute ischemic stroke: An observational prospective study: (TESI study: “Trombolisi/Trombectomia e crisi Epilettiche precoci nello Stroke Ischemico”). Epilepsy Behav..

[125] Bentes C., Martins H., Peralta A.R., Morgado C., Casimiro C., Franco A.C., Fonseca A.C., Geraldes R., Canhao P., Pinho E.M.T. (2017). Epileptic manifestations in stroke patients treated with intravenous alteplase. Eur. J. Neurol..

[126] Tan M.L., Ng A., Pandher P.S., Sashindranath M., Hamilton J.A., Davis S.M., O’Brien T.J., Medcalf R.L., Yan B., Jones N.C. (2012). Tissue plasminogen activator does not alter development of acquired epilepsy. Epilepsia.

[127] Winstein C.J., Stein J., Arena R., Bates B., Cherney L.R., Cramer S.C., Deruyter F., Eng J.J., Fisher B., Harvey R.L. (2016). Guidelines for Adult Stroke Rehabilitation and Recovery: A Guideline for Healthcare Professionals from the American Heart Association/American Stroke Association. Stroke.

[128] Goldstein L.B. (1995). Common drugs may influence motor recovery after stroke. The Sygen In Acute Stroke Study Investigators. Neurology.

[129] Lazar R.M., Fitzsimmons B.F., Marshall R.S., Mohr J.P., Berman M.F. (2003). Midazolam challenge reinduces neurological deficits after transient ischemic attack. Stroke.

[130] Naidech A.M., Kreiter K.T., Janjua N., Ostapkovich N., Parra A., Commichau C., Connolly E.S., Mayer S.A., Fitzsimmons B.F. (2005). Phenytoin exposure is associated with functional and cognitive disability after subarachnoid hemorrhage. Stroke.

[131] Jones F.J.S., Sanches P.R., Smith J.R., Zafar S.F., Hernandez-Diaz S., Blacker D., Hsu J., Schwamm L.H., Westover M.B., Moura L. (2021). Anticonvulsant Primary and Secondary Prophylaxis for Acute Ischemic Stroke Patients: A Decision Analysis. Stroke.

[132] Migdady I., Russman A., Buletko A.B. (2021). Atrial Fibrillation and Ischemic Stroke: A Clinical Review. Semin. Neurol..

[133] Mac Grory B., Flood S., Schrag M., Paciaroni M., Yaghi S. (2019). Anticoagulation Resumption after Stroke from Atrial Fibrillation. Curr. Atheroscler. Rep..

[134] Ruff C.T., Giugliano R.P., Braunwald E., Hoffman E.B., Deenadayalu N., Ezekowitz M.D., Camm A.J., Weitz J.I., Lewis B.S., Parkhomenko A. (2014). Comparison of the efficacy and safety of new oral anticoagulants with warfarin in patients with atrial fibrillation: A meta-analysis of randomised trials. Lancet.

[135] Levine G.N., McEvoy J.W., Fang J.C., Ibeh C., McCarthy C.P., Misra A., Shah Z.I., Shenoy C., Spinler S.A., Vallurupalli S. (2022). Management of Patients at Risk for and with Left Ventricular Thrombus: A Scientific Statement from the American Heart Association. Circulation.

[136] Mentias A., Saad M., Michael M., Nakhla S., Menon V., Harb S., Chaudhury P., Johnston D., Saliba W., Wazni O. (2022). Direct Oral Anticoagulants Versus Warfarin in Patients with Atrial Fibrillation and Valve Replacement or Repair. J. Am. Heart Assoc..

[137] Breithardt G., Baumgartner H., Berkowitz S.D., Hellkamp A.S., Piccini J.P., Stevens S.R., Lokhnygina Y., Patel M.R., Halperin J.L., Singer D.E. (2014). Clinical characteristics and outcomes with rivaroxaban vs. warfarin in patients with non-valvular atrial fibrillation but underlying native mitral and aortic valve disease participating in the ROCKET AF trial. Eur. Heart J..

[138] Woller S.C., Stevens S.M., Kaplan D., Wang T.F., Branch D.W., Groat D., Wilson E.L., Armbruster B., Aston V.T., Lloyd J.F. (2022). Apixaban compared with warfarin to prevent thrombosis in thrombotic antiphospholipid syndrome: A randomized trial. Blood Adv..

[139] Paciaroni M., Agnelli G., Micheli S., Caso V. (2007). Efficacy and safety of anticoagulant treatment in acute cardioembolic stroke: A meta-analysis of randomized controlled trials. Stroke.

[140] Paciaroni M., Agnelli G., Falocci N., Caso V., Becattini C., Marcheselli S., Rueckert C., Pezzini A., Poli L., Padovani A. (2015). Early Recurrence and Cerebral Bleeding in Patients with Acute Ischemic Stroke and Atrial Fibrillation: Effect of Anticoagulation and Its Timing: The RAF Study. Stroke.

[141] Powers W.J., Rabinstein A.A., Ackerson T., Adeoye O.M., Bambakidis N.C., Becker K., Biller J., Brown M., Demaerschalk B.M., Hoh B. (2018). 2018 Guidelines for the Early Management of Patients with Acute Ischemic Stroke: A Guideline for Healthcare Professionals from the American Heart Association/American Stroke Association. Stroke.

[142] Kirchhof P., Benussi S., Kotecha D., Ahlsson A., Atar D., Casadei B., Castella M., Diener H.C., Heidbuchel H., Hendriks J. (2016). 2016 ESC Guidelines for the management of atrial fibrillation developed in collaboration with EACTS. Eur. J. Cardiothorac. Surg..

[143] Kimura S., Toyoda K., Yoshimura S., Minematsu K., Yasaka M., Paciaroni M., Werring D.J., Yamagami H., Nagao T., Yoshimura S. (2022). Practical “1-2-3-4-Day” Rule for Starting Direct Oral Anticoagulants after Ischemic Stroke with Atrial Fibrillation: Combined Hospital-Based Cohort Study. Stroke.

[144] Oldgren J., Asberg S., Hijazi Z., Wester P., Bertilsson M., Norrving B., National T.C. (2022). Early Versus Delayed Non-Vitamin K Antagonist Oral Anticoagulant Therapy after Acute Ischemic Stroke in Atrial Fibrillation (TIMING): A Registry-Based Randomized Controlled Noninferiority Study. Circulation.

[145] Fischer U., Koga M., Strbian D., Branca M., Abend S., Trelle S., Paciaroni M., Thomalla G., Michel P., Nedeltchev K. (2023). Early versus Later Anticoagulation for Stroke with Atrial Fibrillation. N. Engl. J. Med..

[146] Uchino K. (2023). Anticoagulation Conundrum in Acute Ischemic Stroke with Atrial Fibrillation. N. Engl. J. Med..

[147] Crichton S.L., Bray B.D., McKevitt C., Rudd A.G., Wolfe C.D. (2016). Patient outcomes up to 15 years after stroke: Survival, disability, quality of life, cognition and mental health. J. Neurol. Neurosurg. Psychiatry.

[148] Lee K.B., Lim S.H., Kim K.H., Kim K.J., Kim Y.R., Chang W.N., Yeom J.W., Kim Y.D., Hwang B.Y. (2015). Six-month functional recovery of stroke patients: A multi-time-point study. Int. J. Rehabil. Res..

[149] Jorgensen H.S., Nakayama H., Raaschou H.O., Vive-Larsen J., Stoier M., Olsen T.S. (1995). Outcome and time course of recovery in stroke. Part II: Time course of recovery. The Copenhagen Stroke Study. Arch. Phys. Med. Rehabil..

[150] Winters C., van Wegen E.E., Daffertshofer A., Kwakkel G. (2015). Generalizability of the Proportional Recovery Model for the Upper Extremity after an Ischemic Stroke. Neurorehabil. Neural Repair..

